# BedMachine v3: Complete Bed Topography and Ocean Bathymetry Mapping of Greenland From Multibeam Echo Sounding Combined With Mass Conservation

**DOI:** 10.1002/2017GL074954

**Published:** 2017-11-01

**Authors:** M. Morlighem, C. N. Williams, E. Rignot, L. An, J. E. Arndt, J. L. Bamber, G. Catania, N. Chauché, J. A. Dowdeswell, B. Dorschel, I. Fenty, K. Hogan, I. Howat, A. Hubbard, M. Jakobsson, T. M. Jordan, K. K. Kjeldsen, R. Millan, L. Mayer, J. Mouginot, B. P. Y. Noël, C. O'Cofaigh, S. Palmer, S. Rysgaard, H. Seroussi, M. J. Siegert, P. Slabon, F. Straneo, M. R. van den Broeke, W. Weinrebe, M. Wood, K. B. Zinglersen

**Affiliations:** ^1^ Department of Earth System Science University of California Irvine CA USA; ^2^ Bristol Glaciology Centre, School of Geographical Sciences University of Bristol Bristol UK; ^3^ Now at British Geological Survey Nottingham UK; ^4^ Jet Propulsion Laboratory California Institute of Technology Pasadena CA USA; ^5^ Alfred‐Wegener‐Institute, Helmholtz Centre for Polar and Marine Research Bremerhaven Germany; ^6^ Institute of Geophysics University of Texas at Austin Austin TX USA; ^7^ Department of Geography and Earth Science Aberystwyth University Aberystwyth UK; ^8^ Scott Polar Research Institute University of Cambridge Cambridge UK; ^9^ British Antarctic Survey Natural Environment Research Council Cambridge UK; ^10^ Byrd Polar and Climate Research Center Ohio State University Columbus OH USA; ^11^ Centre for Arctic Gas Hydrate, Environment and Climate, Department of Geosciences UiT The Arctic University of Norway Tromsø Norway; ^12^ Department of Geology and Geochemistry Stockholm University Stockholm Sweden; ^13^ Centre for GeoGenetics, Natural History Museum of Denmark University of Copenhagen Copenhagen Denmark; ^14^ Department of Earth Sciences University of Ottawa Ottawa Ontario Canada; ^15^ Department of Geodesy, DTU Space, National Space Institute Technical University of Denmark Kongens Lyngby Denmark; ^16^ Center for Coastal and Ocean Mapping University of New Hampshire Durham NH USA; ^17^ Institute for Marine and Atmospheric Research Utrecht Utrecht University Utrecht Netherlands; ^18^ Department of Geography Durham University Durham UK; ^19^ College of Life and Environmental Sciences University of Exeter Exeter UK; ^20^ Centre for Earth Observation Science, Department of Environment and Geography University of Manitoba Winnipeg Manitoba Canada; ^21^ Greenland Institute of Natural Resources Nuuk Greenland; ^22^ Arctic Research Centre Aarhus University Aarhus Denmark; ^23^ Grantham Institute and Department of Earth Science and Engineering Imperial College London London UK; ^24^ Department of Physical Oceanography Woods Hole Oceanographic Institution Woods Hole MA USA

**Keywords:** Greenland, bathymetry, mass conservation, multibeam echo sounding, radar echo sounding, glaciology

## Abstract

Greenland's bed topography is a primary control on ice flow, grounding line migration, calving dynamics, and subglacial drainage. Moreover, fjord bathymetry regulates the penetration of warm Atlantic water (AW) that rapidly melts and undercuts Greenland's marine‐terminating glaciers. Here we present a new compilation of Greenland bed topography that assimilates seafloor bathymetry and ice thickness data through a mass conservation approach. A new 150 m horizontal resolution bed topography/bathymetric map of Greenland is constructed with seamless transitions at the ice/ocean interface, yielding major improvements over previous data sets, particularly in the marine‐terminating sectors of northwest and southeast Greenland. Our map reveals that the total sea level potential of the Greenland ice sheet is 7.42 ± 0.05 m, which is 7 cm greater than previous estimates. Furthermore, it explains recent calving front response of numerous outlet glaciers and reveals new pathways by which AW can access glaciers with marine‐based basins, thereby highlighting sectors of Greenland that are most vulnerable to future oceanic forcing.

## Key Points

- We present a comprehensive, seamless bed topography across the ice‐ocean margin around Greenland
- Two to 4 times more glaciers have calving fronts grounded below 200 m compared to previous mappings
- Total ice volume of Greenland is 2.99 ± 0.02 times 10^6^ km^3^, yielding a potential sea level rise of 7.42 m, 7 cm greater than previous estimates

## 1 Introduction

Subglacial bed topography and seafloor bathymetry provide fundamental controls on ice dynamics and ocean circulation along Greenland's periphery. The presence of sills in some fjords, for example, can block warm (>2.5°C) subsurface Atlantic water (AW) from interacting with glacier calving fronts, whereas other fjords are too shallow to host AW, or some glacier fronts are grounded above AW levels (e.g., Rignot et al., 2012; Straneo et al., 2010). AW is typically found deeper than 200–300 m below sea level (e.g., Holland et al., 2008; Rignot et al., 2016). When AW reaches glacier termini, calving fronts are exposed to strong ocean‐induced melt, which may be enhanced by subglacial discharge (Bendtsen et al., 2015; Xu et al., 2013). This melt can lead to glacier undercutting, enhanced calving, ice front retreat, flow acceleration, and glacier thinning (Enderlin et al., 2013; Morlighem, Bondizo, et al., 2016; Straneo and Heimbach, 2013). It is therefore critical to determine the locations that are currently exposed to AW and that may be exposed to AW in the future, that is, how far these glaciers need to retreat before the margin reaches higher ground (<200–300 m depth) or becomes land terminating (bed >0 m).

Similarly, subglacial bumps and ridges control the retreat rate of Greenland glaciers and provide temporary stabilizing shoals for grounding lines and ice fronts (e.g., Durand et al., 2011; Morlighem, Bondizo, et al., 2016). It is essential to map these features in sufficient detail, at a spatial resolution compatible with the needs of ocean and ice sheet numerical models (<500 m), to improve both our understanding of ice‐ocean interactions and the predictability of ice sheet evolution in a changing climate using these models (e.g., Aschwanden et al., 2016; Durand et al., 2011; Seroussi et al., 2011).

Following pioneering efforts involving gravity measurements in the 1950s, since the early 1970s bed topography and ice thickness data have been collected primarily by airborne radar sounders that detect the ice/bed interface at nadir, directly beneath the aircraft (Dowdeswell & Evans, 2004; Evans & Robin, 1966). Over the past decade, NASA's Operation IceBridge (OIB) has tripled the amount of ice thickness data (horizontal resolution between 30 and 60 m and vertical resolution ∼10 m), by flying more than 580,000 km of flight tracks over Greenland (Rodriguez‐Morales et al., 2014). These data, combined with other data and a mass conservation approach, have transformed our knowledge of the bed topography of the Greenland ice sheet (Morlighem et al., 2014a).

Despite this wealth of data, bed topography remains challenging to map along the coastal margins because radar‐derived ice thickness data of sufficient quality are lacking in the vicinity of calving fronts, and measurements of fjord bathymetry have often been scarce to nonexistent until recently. Glacier termini are challenging to measure by radar for three reasons: (1) the high density of surface crevasses that reflect off‐nadir radar signal (surface clutter); (2) the ice is warmer, which potentially prevents the radar signal from reaching the bed due to the presence of water pockets; and (3) the deep valleys make the interpretation of radar echograms difficult due to sidewall reflectors (Holt et al., 2006; Jezek et al., 2013). In many cases, no radar‐derived ice thickness measurement is available within 50 km of glacier termini, despite being critical regions for ice sheet models. To map the bed beneath the ice, we have used the mass conservation approach (MC, Morlighem et al., 2011). The accuracy of this mapping method degrades along flow away from radar lines (Morlighem, Rignot, et al., 2016), as the ice moves away from ice thickness constraints. To control the optimization, it is necessary to obtain bathymetry data along the ice fronts (Morlighem, Rignot, et al., 2016).

Here we employ new ocean bathymetry data from 30 different sources, in combination with the mass conservation method to produce a detailed, seamless, and comprehensive map of the bed topography and fjord bathymetry around the entire periphery of Greenland (within 50 km of the coast) at a horizontal spatial resolution of up to 150 m. In poorly charted fjords, we rely on synthetic fjord bathymetries (Williams et al., 2017). We merge the final map with RTopo‐2/IBCAO (Schaffer et al., 2016) at a distance of 50 km away from the coast. We first describe the methodology employed to construct this topographic product, then compare the results with recent bed topography maps from Bamber et al. (2013) and RTopo‐2 (Schaffer et al., 2016), and finally discuss the implications of the results for understanding and modeling the future of Greenland's marine‐terminating glaciers.

## 2 Data and Method

**Figure 1:**
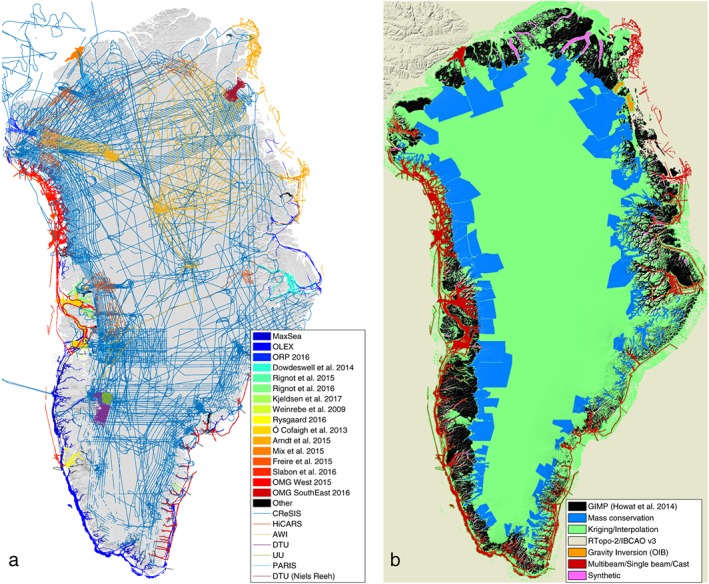
(a) Data coverage, including ice‐penetrating radar measurements (Center for Remote Sensing of Ice Sheets, High CApability Radar Sounder, University of Denmark, Uppsala University, Pathfinder Advanced Radar Ice Sounder, Alfred Wegener Institute) and ocean bathymetry (from single‐beam data in dark blue), and (b) BedMachine v3 bed topography sources, which include mass conservation (MC), kriging, Greenland Ice Mapping Project (GIMP) (Howat et al., 2014), RTopo‐2/IBCAO v3 (Jakobsson et al., 2012; Schaffer et al., 2016), and bathymetry data from multibeam and gravity inversions acquired after the compilation of IBCAO v3.

We compiled radar‐derived ice thickness measurements collected between 1993 and 2016 from the following sources (Figure 1a): NASA's Operation IceBridge, with data processed by the Center for Remote Sensing of Ice Sheets (CReSIS, Leuschen et al., 2010 updated 2016), which is the largest data set with more than 580,000 km of ice thickness measurements; the High CApability Radar Sounder (HiCARS, Peters et al., 2005, 2007), operated by the University of Texas, Institute for Geophysics; the Pathfinder Advanced Radar Ice Sounder (PARIS, Raney, 2010); Alfred Wegener Institute (AWI, Nixdorf et al., 1999); the University of Denmark (DTU, Lindbäck et al., 2014; Thomsen et al., 1997); and Uppsala University (UU, Lindbäck et al., 2014), collected in the vicinity of Russell Gletscher and Nioghalvfjerdsfjorden.

For the ocean bathymetry (Figure 1a), we compiled multibeam echo sounding data (MBES, e.g., Jakobsson et al., 2016) from the following sources: NASA's Oceans Melting Greenland (OMG, OMG Mission, 2016) along the coast of west and southeast Greenland; Slabon et al. (2016) along the Northwest coast; Weinrebe et al. (2009) in Torssukataq and Uummannaq Fjords, for which we also included data from Ó Cofaigh et al. (2013), Dowdeswell et al. (2014), Rignot et al. (2015), Fried et al. (2015), and Rignot et al. (2016); in Melville bay (Freire et al., 2015); and data from the Petermann 2015 Expedition where the Petermann Fjord and the adjacent Hall Basin in Nares Strait were completely mapped with the Swedish icebreaker Oden (Mix et al., 2015). In the region of Disko Bay, we relied on single‐beam and conductivity‐temperature‐depth data from Schumann et al. (2012), and data from Holland et al. (2008) and Straneo et al. (2012) in Illulisat Icefjord. Bathymetry data were also available in the vicinity of Kangerdlussuaq (Sutherland et al., 2014), Nordvestfjord (Dowdeswell et al., 2016), Lille Gletscher (Chauché et al., 2014), Sermilik fjord (Straneo et al., 2016), Godthåbsfjord (Motyka et al., 2017), Sarqardleq fjord (Stevens et al., 2016), Timmiarmiut Fjord, Heimdal Glacier, and Skjoldungen Fjord (Kjeldsen et al., 2017); near the calving front of Bowdoin Glacier (Sugiyama et al., 2015); in Godthåbsfjord (S. Rysgaard, personal communication, 2017), in Young Sound fjord (Rysgaard et al., 2003); near Flade Isblink Ice Cap (Bendtsen et al., 2017); single‐beam data in northwest Greenland from the Ocean Research Project (ORP); and on the continental shelf along the southeast coast (Sutherland & Pickart, 2008). We also added vast amounts of single‐beam data from the Olex seabed mapping system (www.olex.no) as well as crowd‐sourced data from fishing and recreational vessels (MaxSea). While less reliable than MBES data, these data cover an extensive area that significantly improved our mapping in many fjords, where other data were lacking. In the regions of Zachariae‐Isstrøm and Nioghalvfjerdsfjorden, we relied on bathymetry data from Arndt et al. (2015) and bathymetry derived from gravity inversions (Mouginot et al., 2015). We also relied on gravity inversions downstream of Upernavik and Alison's ice fronts. In fjords where coverage is sporadic or nonexistent, we employed the approach proposed by Williams et al. (2017) to construct synthetic yet plausible bathymetry.

To map the subglacial topography of coastal Greenland, we employ a mass conservation approach (MC, Morlighem et al., 2014a, 2013, 2011), which combines ice thickness measurements from radar, high‐resolution (150 m) satellite radar velocity vector data collected in 2008–2009 from Mouginot et al. (2017), surface mass balance reconstruction from RACMO 2.3 downscaled to 1 km averaged for the years 1960–1989 (Noël et al., 2016), and ice thickening/thinning rates from altimetry data differencing between 2003 and 2006 (Khan et al., 2014). These products are provided at a higher resolution and better accuracy than the ones used in BedMachine v2 and significantly improved the mapping in southeast Greenland, where we have less radar data to constrain the calculation. Morlighem et al. (2014a) provide a complete description of the MC method.

For some glaciers, bathymetry measurements are available within a few kilometers of the glacier calving fronts. These data are used to provide additional constraints to MC in places not well covered with radar data. We use here the approach described in Morlighem, Rignot, et al. (2016), where bathymetry data are included in the cost function to be minimized:

(1) $$J(H)=\underset{T}{\int }\frac{1}{2}{\left(H-H_{\text{obs}}\right)}^{2}dT+\underset{\text{constraint from bathymetry data}}{\underset{︸}{\underset{\text{terminus}}{\int }\frac{1}{2}{\left(H-\left(s-b\right)\right)}^{2}ds}}+R(H)$$

where *H* is the modeled ice thickness that satisfies the conservation of mass; *H*_obs_ is the measured ice thickness data measured along flight lines, *T*; and R is a regularizing term to avoid unrealistic spurious oscillation in ice thickness due to overfitting. The second term of equation (1) forces the MC ice thickness to be as close as possible to the ice front thickness estimated by subtracting the surface elevation, *s* (Howat et al., 2014), from the ocean bathymetry, *b*, at the terminus. For 70% of the glaciers that were remapped using the bathymetry constraint, we found that the bed estimate from BedMachine v2 (that was not constrained by bathymetry data) was within 100 m of bathymetry measurements, which was less than our error estimate (Table S1 in the supporting information).

The regions in blue in Figure 1b show that MC is applied mainly along the coast where ice velocities are higher than 50 m/yr, which are regions where MC is most reliable (Morlighem et al., 2014b). Since the release of BedMachine v2, we have increased the surface area mapped with MC by 44%, primarily along the east coast. We then stitch together all individual MC ice thickness maps. Two adjacent MC maps of ice thickness are constrained by the same flight lines along their boundaries to ensure a seamless transition between adjacent maps. We create a gap of ∼ 2 km between the two maps and use a simple interpolation (inverse distance weighting) in order to create a smooth transition.

Away from the regions of fast flow, we map the ice thickness in the interior of the ice sheet using kriging (Deutsch & Journel, 1997). Kriging is applied 5 km away from the MC maps, and we include all MC ice thickness results as additional point measurements to the kriging.

We deduce the subglacial topography by subtracting our ice thickness map from a surface digital elevation model from the Greenland Ice Mapping Project (GIMP) (Howat et al., 2014), which has a nominal date of 2007. This bed topography is then combined with all bathymetry data in the fjords and a natural neighbor interpolation along the fjords (Figure 1b). In uncharted or poorly charted fjords, we use a synthetic fjord method (Williams et al., 2017) that enforces a parabolic across‐flow profile consistent with the bed depth at glacier termini and guided by available bathymetry measurements. We merge this map with RTopo‐2 (Schaffer et al., 2016) 50 km away from the coast. RTopo‐2 is mostly based on the International Bathymetric Chart of the Arctic Ocean v3.0 (IBCAO, Jakobsson et al., 2012), except in northeast Greenland where RTopo‐2 uses the bathymetric compilation by Arndt et al. (2015).

**Figure 2:**
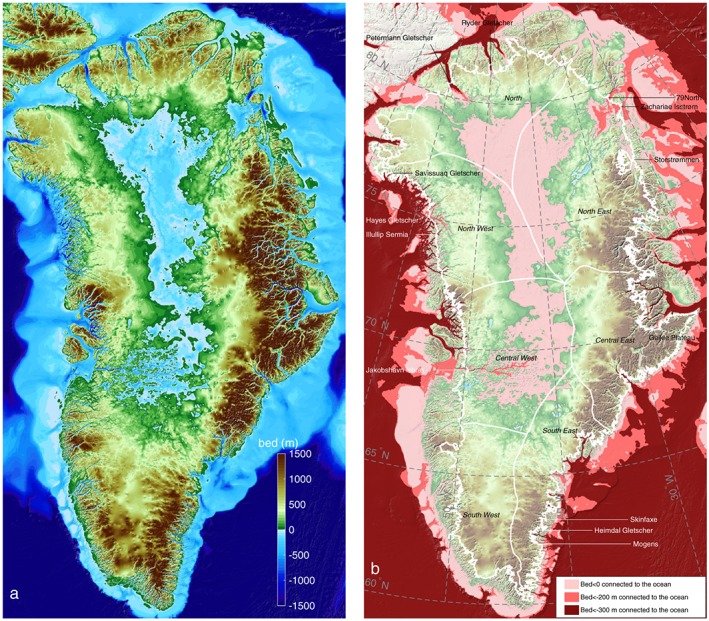
(a) BedMachine v3 bed topography (m), color coded between −1500 m and +1500 m with respect to mean sea level, with areas below sea level in blue and (b) regions below sea level (light pink) that are connected to the ocean and maintain a depth below 200 m (dark pink) and that are continuously deeper than 300 m below sea level (dark red). The thin white line shows the current ice sheet extent.

The final bed topography map is assembled on a grid with a horizontal resolution of 150 m (using a Polar Stereographic North projection, with a central meridian of 45°W and standard parallel of 70°N), but the true horizontal resolution of the bed topography depends on the source data used to create the maps in that region. It is about 1,000 m in regions where kriging, RTopo‐2, and gravity inversions are used, 400 m in the regions mapped with MC, and 150 m for the regions of ice‐free land or the regions for which we have MBES bathymetry data (Figure 1b), as these data are provided at a higher resolution than our 150 m grid. Figure 2a shows the final bed topography product, with seamless transitions across glacier calving fronts.

All the data sets used to reconstruct the ice thickness cover the period 2003–2008, except the observed ice thickness data that cover a longer period: 1993–2016. While many glaciers along the coast have been thinning, the cumulative amount of thinning remains less than the uncertainty in radar‐derived ice thickness measurement (∼50 m) except in coastal regions that experience strong thinning.

## 3 Results and Discussion

Accounting for scale corrections (due to the polar stereographic projection which is increasingly distorted with distance from the defined latitude of true scale, 70°N), this new bed topography yields a total ice volume of 2.99 ± 0.02 10^6^ km^3^ (or 2.74 ± 0.02 10^6^ Gt, assuming an ice density of 916.7 kg m^−3^). The volume above floatation, which would directly contribute to sea level rise if the Greenland ice sheet were to melt entirely, is 2.93 ± 0.02 10^6^ km^3^ (or 2.69 ± 0.02 10^6^ Gt). Assuming that the density of the ice is 916.7 kg m^−3^, for an average density of sea water of 1,027 kg m^−3^, and assuming that 361.8 Gt of ice contributes to 1 mm of global eustatic sea level rise, this new map suggests that the Greenland ice sheet has a total sea level rise potential of 7.42 ± 0.05 m, which is 7 cm larger than stated by Stocker et al. (2013).

**Figure 3:**
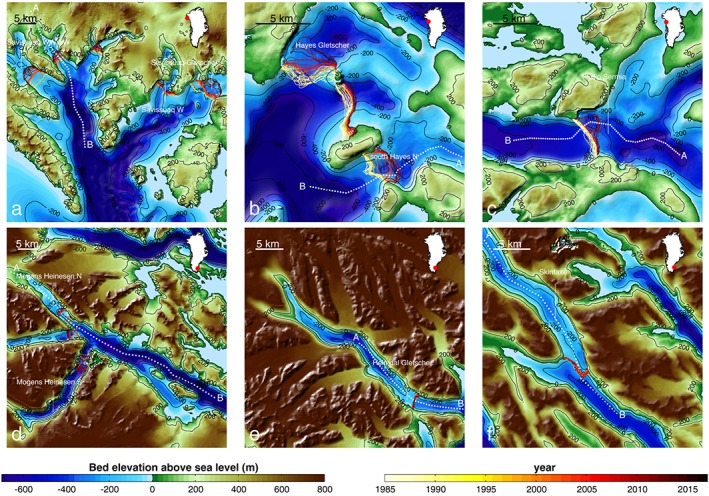
Bed topography for different sectors of Greenland: (a) the region of Savissuaq Gletscher, (b) Hayes Gletscher, (c) Illullip Sermia, (d) Mogens Heinesen N, (e) Heimdal Gletscher, and (f) Skinfaxe. The yellow/red lines indicate the ice front position between 1985 and today from Landsat data, and the white dotted line shows the profile used in Figure 4. The topography is color coded between −700 m and 800 m, and contours are shown every 200 m from −800 m to 200 m above sea level. Some glaciers, such as the one 10 km northwest of Heimdal Gletscher, were not mapped using MC.

**Figure 4:**
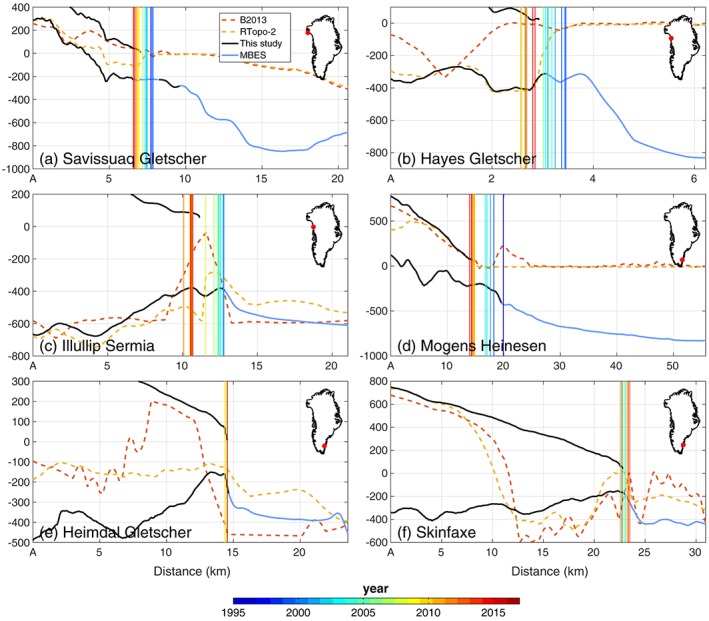
Surface and bed topography along six profiles (see white dotted lines in Figure 3) from this study (solid black) and bed from B2013 (dotted red, Bamber et al., 2013) and RTopo‐2 (dotted yellow, Schaffer et al., 2016). Multibeam bathymetry data (MBES) are shown in blue. The vertical lines show the ice front position between 1995 and today.

Figure 3 illustrates some examples of bed mapping across calving fronts, and Figure 4 shows the surface and bed elevations compared with two recently released products: B2013 (Bamber et al., 2013) and RTopo‐2 (Schaffer et al., 2016) along some cross sections. B2013 relies solely on kriging for the bed topography under the ice sheet and the IBCAO v3 (Jakobsson et al., 2012) for the ocean bathymetry. In a number of fjords, no bathymetry data were available prior to OMG and B2013 manually lowered the bathymetry from IBCAO in order to better represent deep fjords. RTopo‐2 relied on mass conservation products from BedMachine v2 (Morlighem et al., 2014a) for grounded ice and IBCAO for the ocean bathymetry in this region. Ice front positions were derived from Landsat 5, 7, and 8 data.

In the region of Savissuaq Gletscher, in northwest Greenland, the new bathymetry data from OMG reveal fine‐scale (<1 km) details, such as moraines that may originate from the Little Ice Age (Figure 3a) and may have acted as pinning points as the glacier retreated inland. The topography revealed by OMG is significantly deeper than in previous mapping (>600 m below sea level) and rises gradually toward the ice margin. In this region, the bed from BedMachine v2, which is included in RTopo‐2 (Figure 4a), showed a misfit with OMG data of about 100 m. This is one of the places where accounting for bathymetry data significantly improves the mapping of bed topography upstream. The shape of the bed between BedMachine v2 and v3 did not change significantly but shifted down by 100 m near the margin. The original offset, which is less than our error estimate, may be due to several factors such as an underestimated thinning rate or underestimated accumulation. We observe that the ice front has been retreating over a relatively flat bed (Figure 4a) but is now entering a region of prograding bed slopes (i.e., the bed rises inland), which we expect will lead to slowing down of retreat.

In the vicinity of Hayes Gletscher, in northwest Greenland (Figures 3b and 4b), new bathymetry data again reveal a topography that is significantly different from previous mappings: in the south side of the domain, the bed remains deep below sea level (< 800 m) and rises to about 200 m below sea level. The ice front of Hayes was stabilized by a sill of about 200 m but was dislodged from this sill in 2005 and retreated into a region of retrograde bed (i.e., the bed deepens inland) (Post, 1975; Weertman, 1974). According to our mapping, the ice front will retreat another 3 km before the bed becomes prograde again. South Hayes N was also stable on a sill until 1998 when it started to retreat inland, where the bed is deeper. It is now resting on the deepest part of the bed, and we expect the ice front to either stabilize at its current position or retreat at a slower pace into an area of prograde slope.

Illullip Sermia (Figures 3c and 4c) has also been resting on a pronounced sill of about 300 m where it has been stable since at least 1985, but over the past 5 years, the northern side of the calving front has retreated by about 2 km inland where the bed is deepest. The asymmetry of the retreat is in agreement with our new bed mapping and suggests that this glacier will continue to retreat for at least another 5 km, where the bed is strongly retrograde.

In the three following examples in southeast Greenland (Figures 3d–3f), we did not have reliable ice thickness measurements to correctly constrain the bed depth in BedMachine v2. Eastern Greenland is on average ∼1,000 m higher than western Greenland, and glaciers have carved their way through the mountain ranges by glacial erosion over many glacial cycles (Kessler et al., 2008), forming deep, highly entrenched valleys. The glaciers flowing through these fjords are particularly difficult to sound due to their complex geometry. Applying MC in these fjords is therefore challenging since the approach is poorly constrained. Prior to OMG, very few glaciers in the southeast could be mapped using MC. With new bathymetry measurements at the calving face of some glaciers of southeast Greenland, we now have the essential constraint at the ice front to make MC mapping reliable.

In the region of Mogens Heinesen (Figures 3d and 4d), OMG data reveal a deep (800 m below sea level) fjord that rises rapidly close to calving fronts, which are resting on a prograde slope. All three glaciers have been retreating over the past two decades, but their rate of retreat seems to be slowing down as the grounding line migrates to shallower topography.

Heimdal Gletscher and Skinfaxe glacier (Figures 3e, 3f, 4e, and 4f) are sitting on ledges within their fjord, and their fronts have been stable for the past 10 years. There is evidence that Skinfaxe glacier has been stable at that position since at least the 1930s (Bjørk et al., 2012), which suggests that significant thinning is required to dislodge these glaciers from their current position. The glacier bed remains below sea level several tens of kilometers inland. Similar deep fjords are found to be widespread in southeast Greenland.

These narrow and deep fjords have important implications for the current and future state of the Greenland ice sheet as they can provide pathways for AW to interact with glacier termini. To investigate the regions that are in contact with the ocean, we determine locations that are continuously below sea level from the continental shelf to the ice sheet bed (light pink area in Figure 2b). As glaciers around Greenland retreat, these regions will remain in contact with the ocean. We also determine the regions that are continuously below a depth of 200 and 300 m, respectively, and are currently connected to the ocean below these depths (pink and dark red areas in Figure 2b, respectively). Glaciers that retreat within these regions will potentially remain in contact with warm AW as they do so. Since submarine bed channels are widespread and extend far inland, these glaciers will remain vulnerable to ocean warming as they retreat for hundreds of kilometers.

This map suggests that while south and east Greenland are not significantly vulnerable to AW as the bed topography is mainly above sea level in these regions, west Greenland and major glaciers in northern Greenland are currently exposed to AW and will remain exposed for tens to hundreds of kilometers as they retreat inland. Among the 243 glaciers listed in Rignot and Mouginot (2012), we find that 139 of them are marine terminating (81 in B2013 and 129 in RTopo‐2), 28 have pathways to the ocean that remain below 300 m below sea level (22 in B2013 and 7 in RTopo‐2, Table S1), and 67 are exposed to ocean waters that remain below 200 m below sea level (32 in B2013 and 17 in RTopo‐2, Table S1). This new bed shows that between 30 and 100% more glaciers are potentially exposed to AW than suggested by previous mapping, which represents ∼55% of the ice sheet's total drainage area (45% in B2013 and 35% in RTopo‐2). The Greenland ice sheet is therefore more exposed to ocean warming than understood previously.

Although northwest Greenland is particularly well surveyed in terms of ice thickness measurements, east Greenland remains underconstrained in many places, and the coast between Storstrømmen and north of the Geikie Plateau must be further surveyed. These improvements would allow us to run MC in some regions where there are not enough measurements to properly constrain the model. Many lines have been flown south of the Geikie Plateau along the coast, but the bed remains challenging to sound. More gravity data could help better constrain the topography if MBES mapping is not possible. Many fjords along the coast also remain to be mapped (Table S1), especially in regions where we are relying on synthetic bathymetry (pink in Figure1b). Additional bathymetry measurements in the fjords and on the continental shelf will further improve the reliability and quality of BedMachine.

This mapping provides a physically based description of the subglacial topography and ocean bathymetry in the vicinity of the Greenland ice sheet. The product makes it possible to consider modeling ocean circulation in Greenland fjords. It also offers major improvements in the robustness of ice sheet model predictions, as it includes many features that may play an important role on ice dynamics as glacier calving fronts continue to retreat. As more radar, gravity, or multibeam data become available, we will update the map to continuously improve projections of the contribution of the Greenland ice sheet to sea level rise. The final product will be available as an Operation IceBridge Earth Science Data Set at the National Snow and Ice Data Center (NSIDC).

## 4 Conclusion

We present a new high‐resolution bed map of the Greenland ice sheet and ocean bathymetry, with a seamless transition across glacier termini. The map combines mass conservation and newly collected ocean bathymetry data from OMG and includes bathymetric data from previous marine surveys. Our new estimate for Greenland's sea level rise potential is 7 cm higher than the previously published value, and we find that between 30 and 100% more glaciers are potentially exposed to AW than suggested by RTopo‐2 or B2013. We also find that our new map is consistent with the pattern of ice front retreat along the coast, where the glaciers that have been retreating the most were resting on retrograde beds. This map is a self‐consistent data set that opens the door to high‐resolution coupled ice sheet and ocean numerical modeling, which should ultimately improve our ability to model the evolution of the Greenland ice sheet in a changing climate.

## Supporting information



Supporting Information S1

Table S1
