# Supplementary material for: BedMachine v3: Complete Bed Topography and Ocean Bathymetry Mapping of Greenland From Multibeam Echo Sounding Combined With Mass Conservation
Source: Geophys Res Lett. 2017 Nov 1;44(21):11051–61. doi: 10.1002/2017GL074954 (PMC5726375; doi:10.1002/2017GL074954)
Supplement: Supplementary file 1 — Supporting Information S1 [file GRL-44-11051-s001.pdf]

Supplementary Information for

“BedMachine v3: Complete bed topography and ocean bathymetry mapping of Greenland from multi-beam radar sounding combined with mass conservation”

M. Morlighem[1], C. N. Williams[2,3], E. Rignot[1,4], L. An[1], J. E. Arndt[5], J. L. Bamber[2], G. Catania[6], N. Chauché[7], J. A. Dowdeswell[8], B. Dorschel[5], I. Fenty[4], K. Hogan[9], I. Howat[10], A. Hubbard[7,11], M. Jakobsson[12], T. M. Jordan[2], K. K. Kjeldsen[13,14,15], R. Millan[1], L. Mayer[16], J. Mouginot[1], B. P. Y. Noël[17], C. Ó Cofaigh[18], S. Palmer[19], S. Rysgaard[20,21,22], H. Seroussi[4], M. J. Siegert[23], P. Slabon[5], F. Straneo[24], M. R. van den Broeke[17], W. Weinrebe[5], M. Wood[1] and K. B. Zinglersen [21]

[1]University of California, Irvine, Department of Earth System Science, 3218 Croul Hall, Irvine, CA 92697-3100, USA
[2]Bristol Glaciology Centre, School of Geographical Sciences, University of Bristol, Bristol, UK
[3]Now at British Geological Survey, Nicker Hill, Keyworth, Nottingham, NG12 5GG, UK
[4]Jet Propulsion Laboratory - California Institute of technology, 4800 Oak Grove Drive, Pasadena, CA 91109-8099, USA
[5]Alfred-Wegener-Institute, Helmholtz Centre for Polar and Marine Research, Bremerhaven, Germany
[6]Institute of Geophysics, University of Texas, Austin, USA
[7]Department of Geography and Earth Science, Aberystwyth University, Aberystwyth, UK
[8]Scott Polar Research Institute, University of Cambridge, Cambridge CB2 1ER, UK
[9]British Antarctic Survey, Natural Environment Research Council, High Cross, Madingley Road, Cambridge CB3 0ET, UK
[10]Byrd Polar and Climate Research Center, Ohio State University, Columbus, OH, USA
[11]Centre for Arctic Gas Hydrate, Environment and Climate, Department of Geosciences, UiT The Arctic University of Norway, 9037 Tromsø, Norway
[12]Department of Geology and Geochemistry, Stockholm University, Stockholm, Sweden
[13]Centre for GeoGenetics, Natural History Museum of Denmark, University of Copenhagen, Copenhagen, DK-1350, Denmark
[14]Department of Earth Sciences, University of Ottawa, Ottawa, K1N 6N5, Canada
[15]Department of Geodesy, DTU Space, National Space Institute, Technical University of Denmark, Kongens Lyngby, Denmark
[16]Center for Coastal and Ocean Mapping, University of New Hampshire, NH 03824, USA
[17]Institute for Marine and Atmospheric research Utrecht (IMAU), Utrecht University, the Netherlands
[18]Department of Geography, Durham University, Science Laboratories, South Road, Durham DH1 3LE, UK
[19]College of Life and Environmental Sciences, University of Exeter, Exeter EX4 4RJ, UK
[20]Centre for Earth Observation Science, Department of Environment and Geography, University of Manitoba, Winnipeg, Canada
[21]Greenland Institute of Natural Resources, Nuuk, Greenland
[22]Arctic Research Centre, Aarhus University, Aarhus, Denmark
[23]Grantham Institute and Department of Earth Science and Engineering, Imperial College, London SW7 2AZ, UK
[24]Department of Physical Oceanography, Woods Hole Oceanographic Institution, Woods Hole, Massachusetts, USA

correspondence: mathieu.morlighem@uci.edu

Contents of this file

1. Text S1
2. Text S2
3. Text S3
4. Text S4
5. Figure S1
6. Figure S2

7. Figure S3
8. Figure S4
9. Figures S5, S6 and S7
10. Figures S8, S9 and S10

Additional Supporting Information (Files uploaded separately)

1. Table S1

Introduction

This supporting information provides additional details about BedMachine v3: what is included in the NetCDF file, the kriging parameters used in the interior of the ice sheet, and some details about the method to produce synthetic bathymetry.

Text S1: BedMachine v3 dataset

The data will be available in one single file in NetCDF format (2.1G Gb) and all heights are in meters above mean sea level (the geoid used is provided in the NetCDF file). All the data use the same 150 m-resolution grid although the "true" resolution of the bed topography and ice thickness varies depending on the method used and the input data. This dataset uses data from 1993 to 2016 and has a nominal date of 2007 (same as the GIMP DEM from *Howat et al.* [2014]). The following fields are provided:

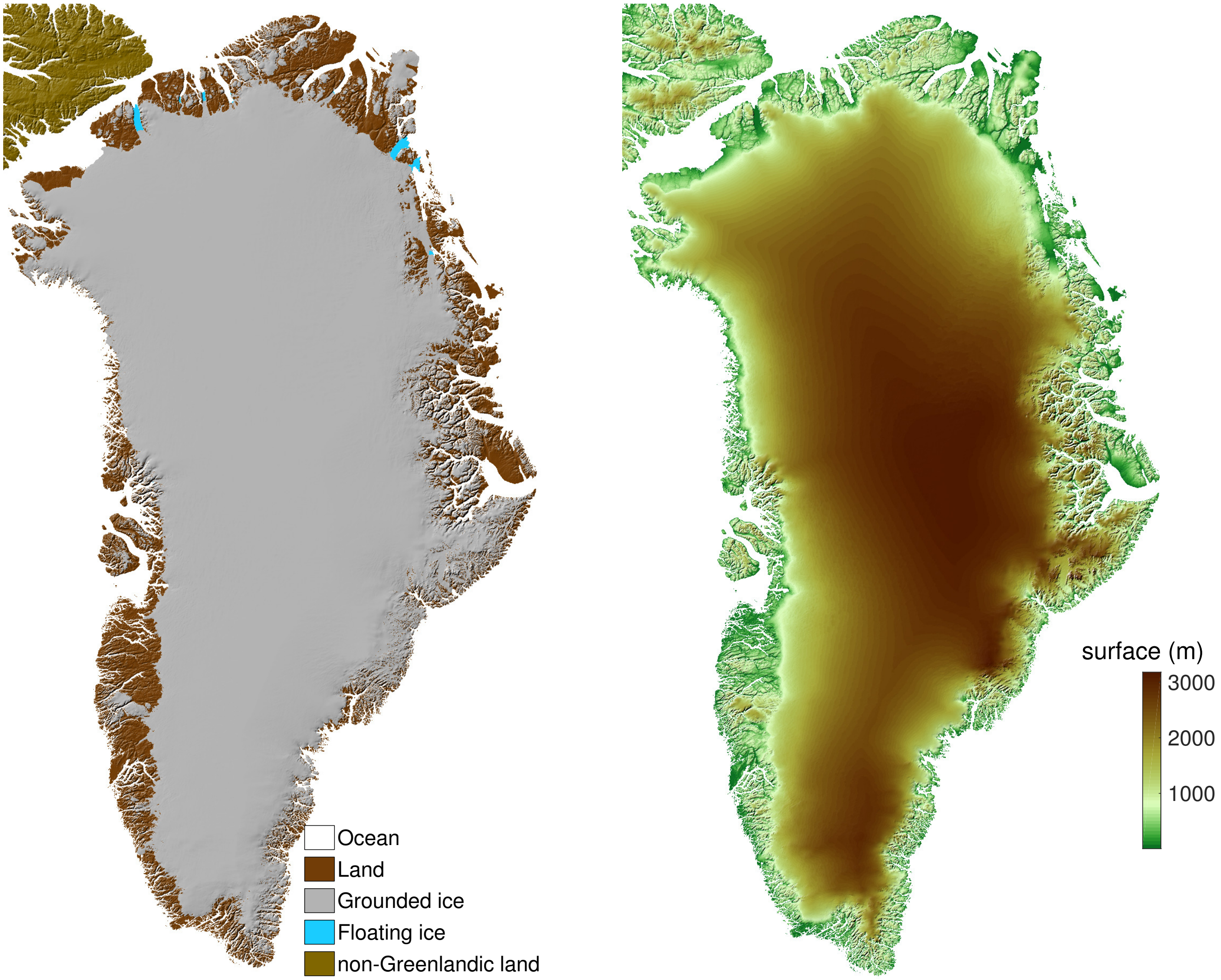

Figure S1: Left: The ice/land/ocean masks are from *Howat et al.* [2014], and the floating ice is derived from InSAR grounding lines. Right: Surface dem is from *Howat et al.* [2014]

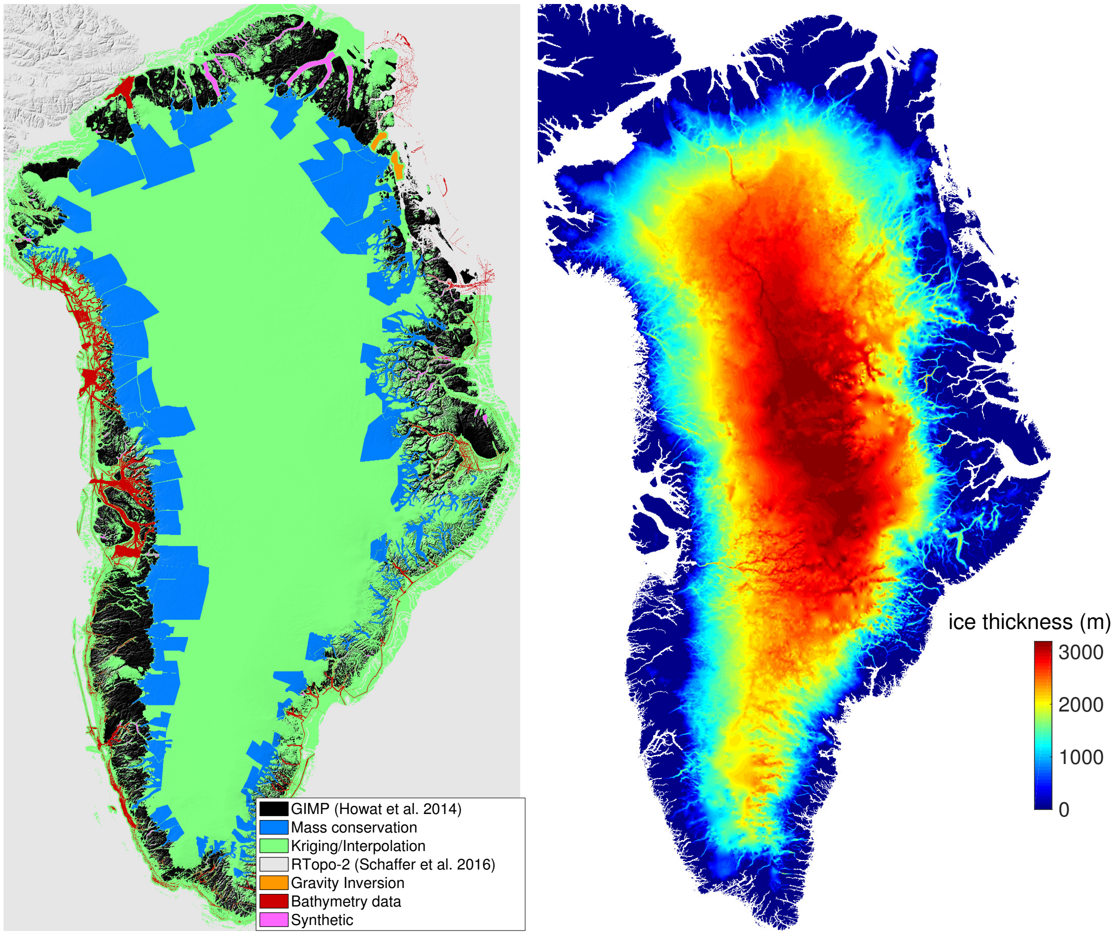

Figure S2: Left: Methods used to compute the bed topography. Right: Ice thickness

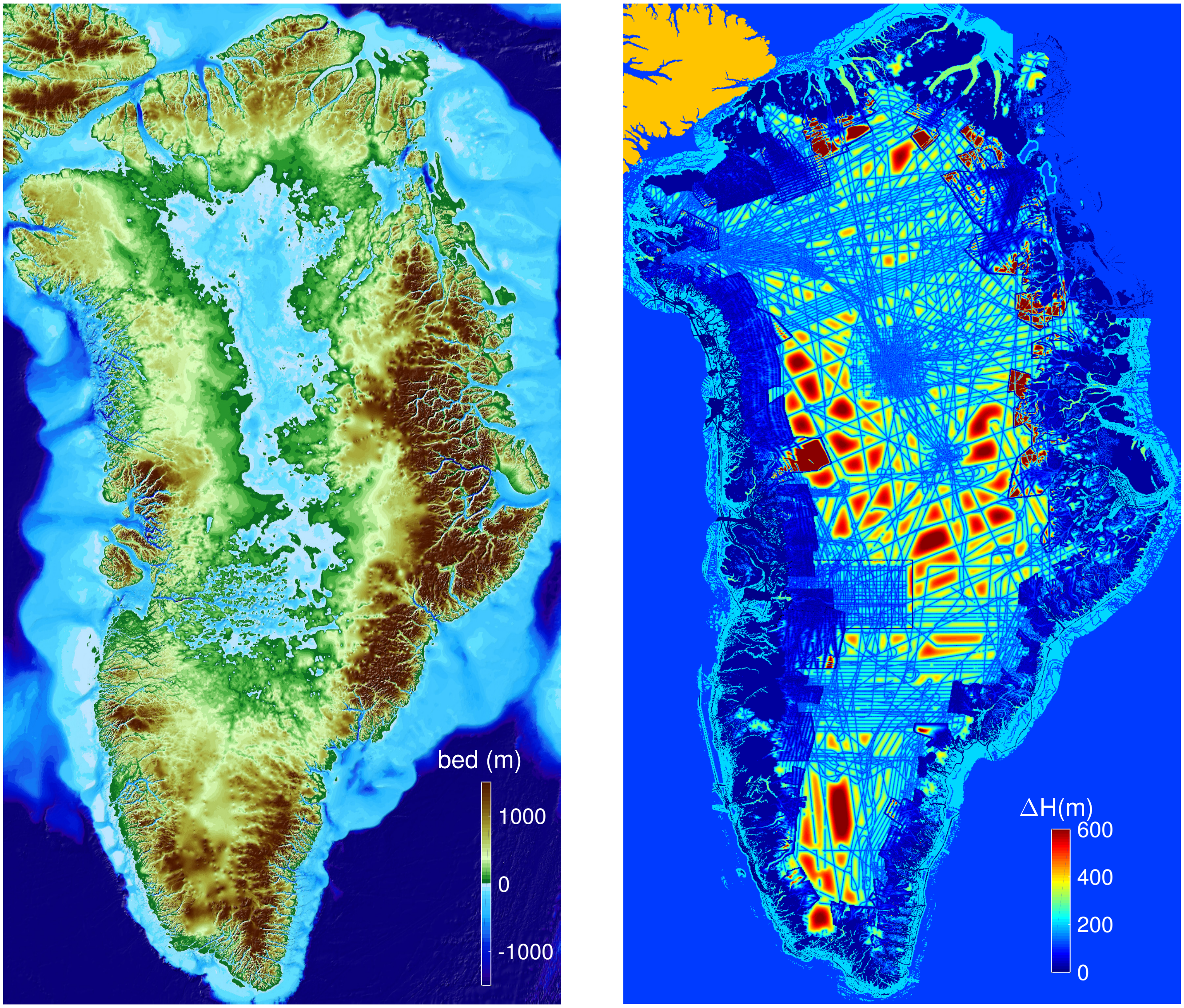

Figure S3: Left: Bed topography. Right: Bed topography error margin

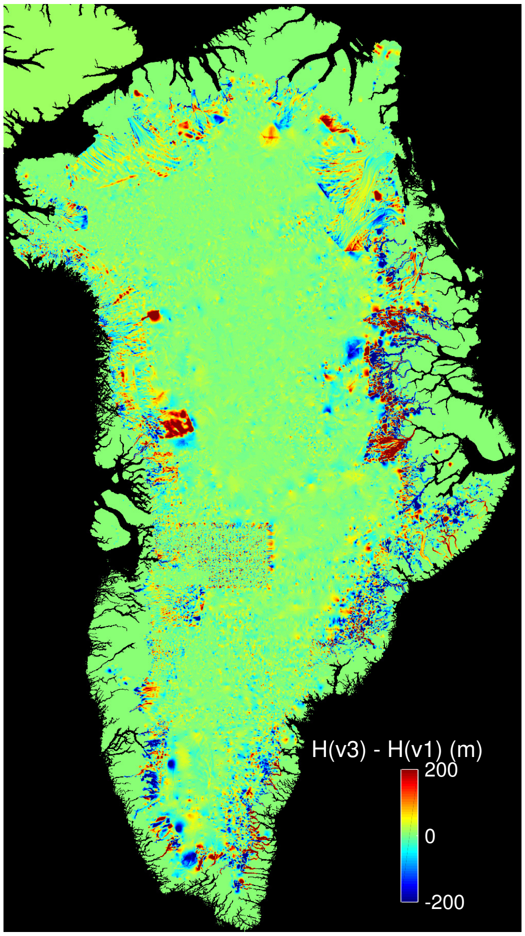

Figure S4: Difference between ice thickness from BedMachine v3 and v1

Text S2: Kriging parameters

For the kriging in the interior of the ice sheet, we used the kriging algorithm that is part of the Ice Sheet System Model ISSM. The variogram is modeled as a Gaussian function, with a sill of 100 m, a range of 8 km and a nugget effect of 50 m, to account for uncertainty in ice thickness measurements.

Text S3: Synthetic fjord bathymetry

Due to its sinuous and anisotropic nature, the mapping of fjord bathymetry is treated separately from bathymetry further from the Greenland coast. On a case-by-case basis, where a fjord - or part of a fjord - has limited observational coverage, it is assigned a geospatial network structure by mapping nodes along its centerline and across its width using the synthetic fjord bathymetry method described in [*Williams et al.*, 2017], available here `https://zenodo.org/record/827347` (DOI:10.5281/zenodo.827346), and as described below. As an approximation, fjords are assumed to adopt a parabolic ("U-shaped") cross-sectional profile geometry that propagates along their length. Elevations at either end of the fjord centerline are taken from the nearest prior constrained elevations. At the fjord head, this includes bed elevations from mass conservation, and at the fjord mouth this includes the nearest bathymetric observations. Where the synthetic method is applied, each parabolic fjord cross section is determined by three declared elevations. At the fjord edges these are extracted from ice free altimetry [*Howat et al.*, 2014]

and across the fjord's width, any available observations are used. If observations are available along the fjord length, this ensures the synthetic profile incorporates those measurements. Elevations are then assigned along the entire centerline by linear interpolation between points. In the worst case, this linear interpolation will be between only 2 points - the head and mouth of the fjord. The imposition of this synthetic structure provides a series of points which can then be integrated into the overall DEM gridding procedure, removing reliance on interpolation procedures to predict likely fjord structure in regions of poor observational coverage. An example of the impact of the synthetic bathymetry method is illustrated in figure S5, comparing B2013 to BedMachine v3. Of particular note is that the synthetic fjord method leads to a reduced overestimation of depths than previous bed/bathymetry datasets.

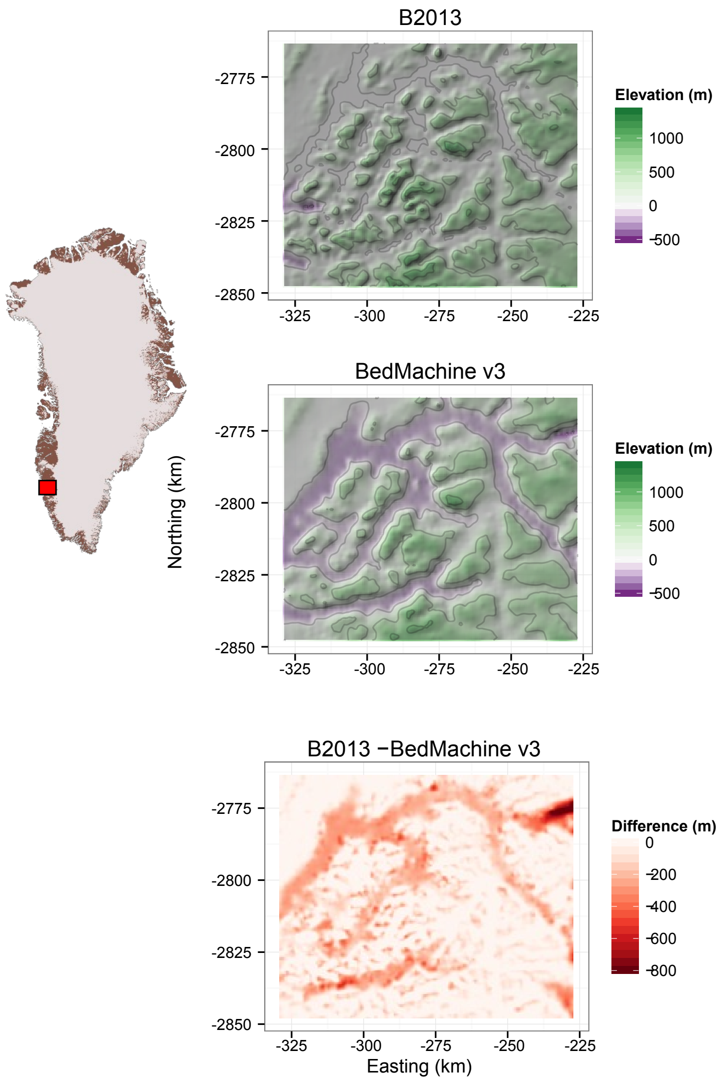

Figure S5: Bed topography of Godthåbsfjord from B2013 [*Bamber et al.*, 2013], and this new study, which includes synthetic bathymetry in the fjord.

Text S4: Examples of mass conservation

We illustrate here how MC optimizes the input data to best fit ice thickness measurements over the region of Upernavik in Northwest Greenland, and Helheim in Southwest Greenland. The first figure (Fig. S6) shows the initial input data and the resulting calculated ice thickness, i.e., we take the input data as they are and compute the ice thickness by solving:

$$\left\{ \begin{array}{rcll} \nabla \cdot H\bar{\mathbf{v}} & = & \dot{a} & \text{in } \Omega \\ H & = & H_{obs} & \text{on } \Gamma_- \end{array} \right. \tag{1}$$

where H is the ice thickness, $\bar{\mathbf{v}}$ is the depth-averaged ice velocity vector, $\dot{a}$ is the apparent mass balance (i.e. surface mass balance minus rate of thickness change).

In figure S7, we show the same fields but after optimization: we change the input data ($\bar{\mathbf{v}}$ and $\dot{a}$) in order to minimize the misfit between the calculated ice thickness H, and IPR derived ice thickness measurements. Here, we allow the velocity to decrease by up to 5% (since $\bar{\mathbf{v}}$ might be smaller than surface velocity due to vertical shear), and the apparent mass balance is allowed to change by up to ± 2 m/a to account for error in surface mass balance or thinning rates. Our control spaces are therefore:

$$\dot{a} \in \{\dot{a}_{obs} + \alpha \text{ m/a}, \quad \alpha \in [-2\ 2]\} \tag{2}$$

$$\overline{v}_x \in \left\{\alpha_1 v_x^{obs} + \alpha_2 \text{ m/a}, \quad \alpha_1 \in [0.95\ 1]\,,\ \alpha_2 \in [-50\ 50]\right\} \tag{3}$$

$$\overline{v}_y \in \left\{\alpha_1 v_y^{obs} + \alpha_2 \text{ m/a}, \quad \alpha_1 \in [0.95\ 1]\,,\ \alpha_2 \in [-50\ 50]\right\} \tag{4}$$

Finally, figure S8 shows the difference between the original input data and the optimized output. Figures S9, S10 and S11 show the same fields on Helheim.

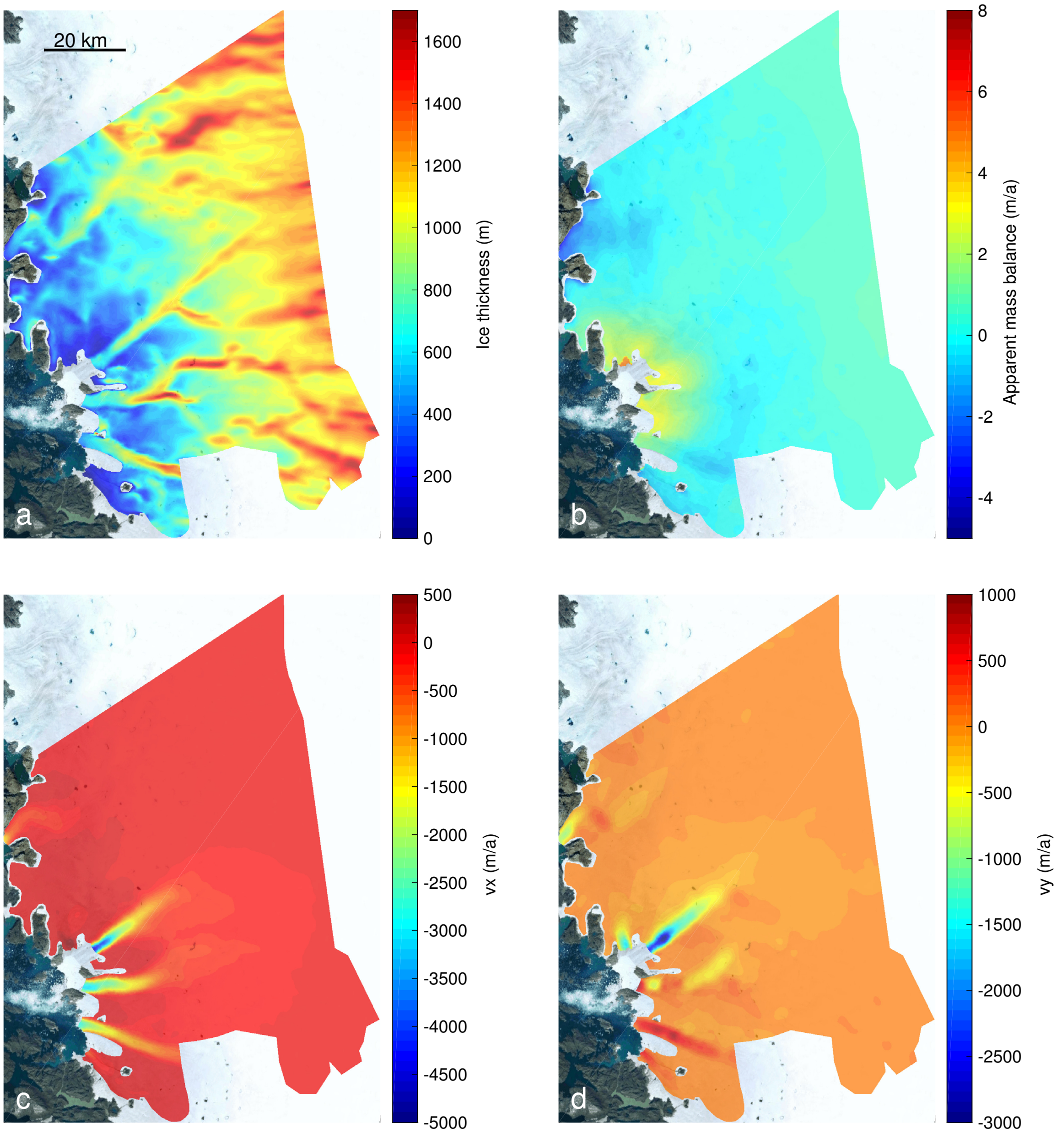

Figure S6: (a) MC calculated ice thickness before optimization, (b) initial apparent mass balance, (c) observed surface x-component of the ice velocity, (d) observed surface y-component of the ice velocity.

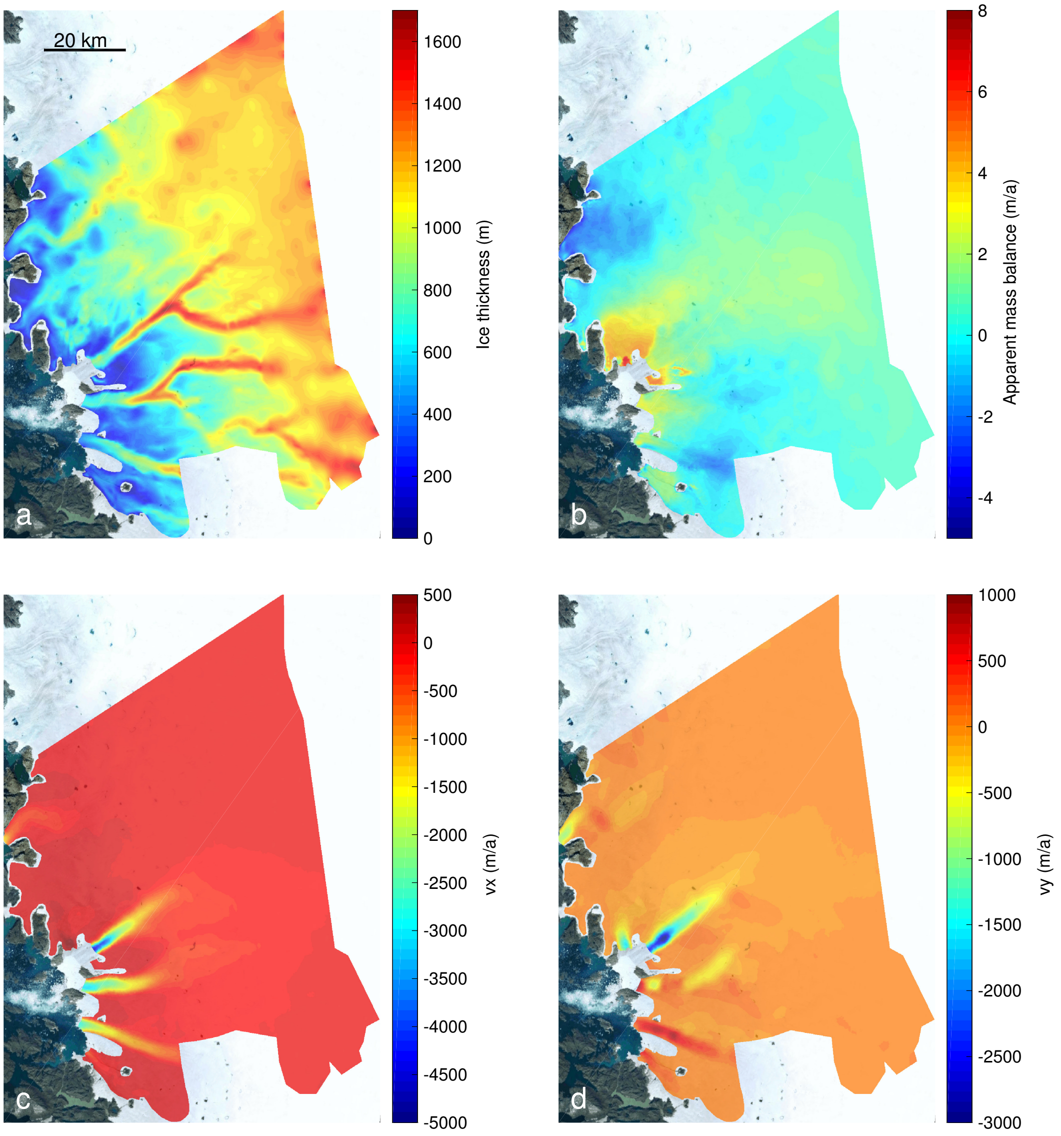

Figure S7: (a) MC calculated ice thickness after optimization, (b) optimized apparent mass balance, (c) optimized depth averaged x-component of the ice velocity, (d) optimized depth averaged y-component of the ice velocity.

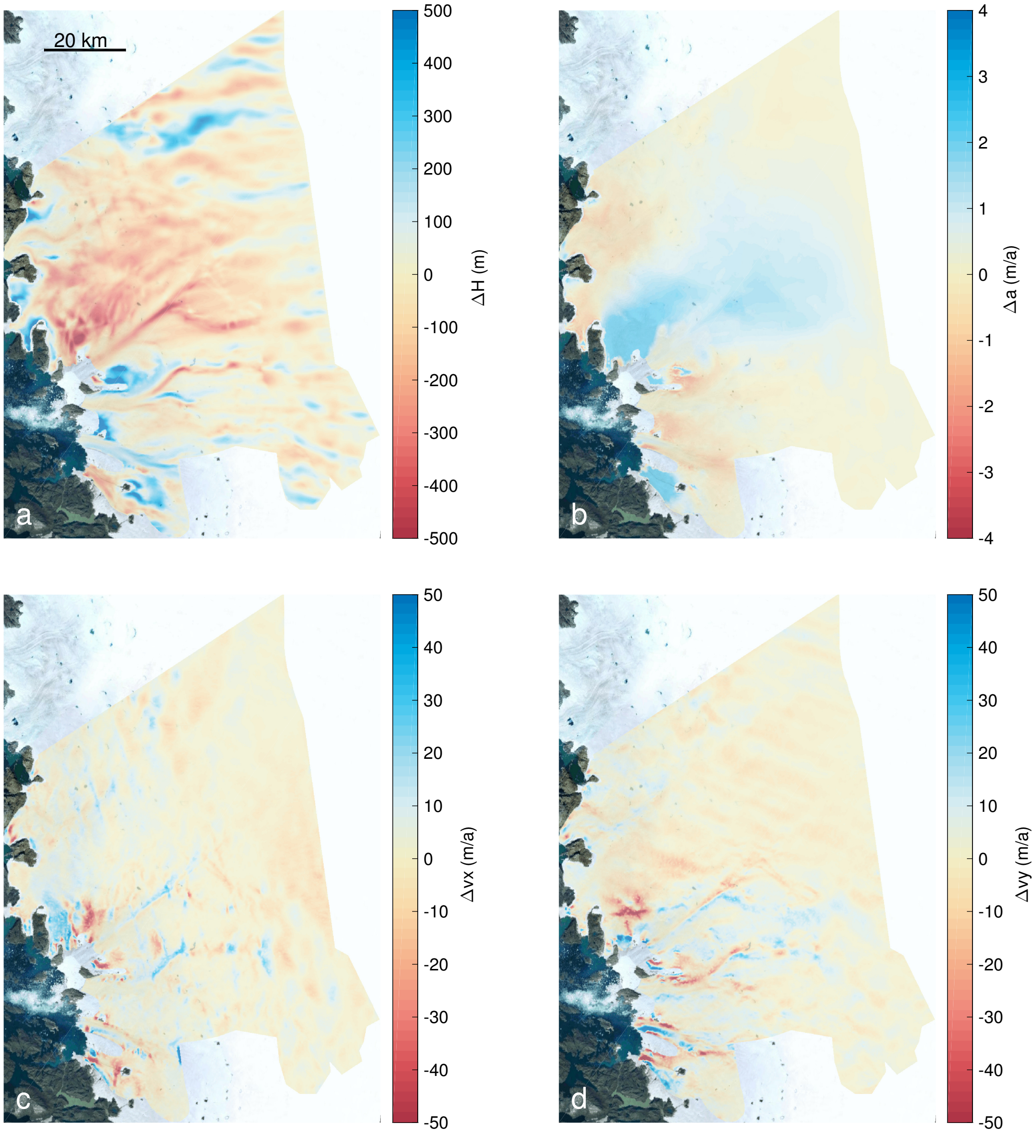

Figure S8: (a) difference between initial and optimized ice thickness, (b) difference between initial and optimized apparent mass balance, (c) between initial and optimized $\bar{v}_x$, (d) between initial and optimized $\bar{v}_y$.

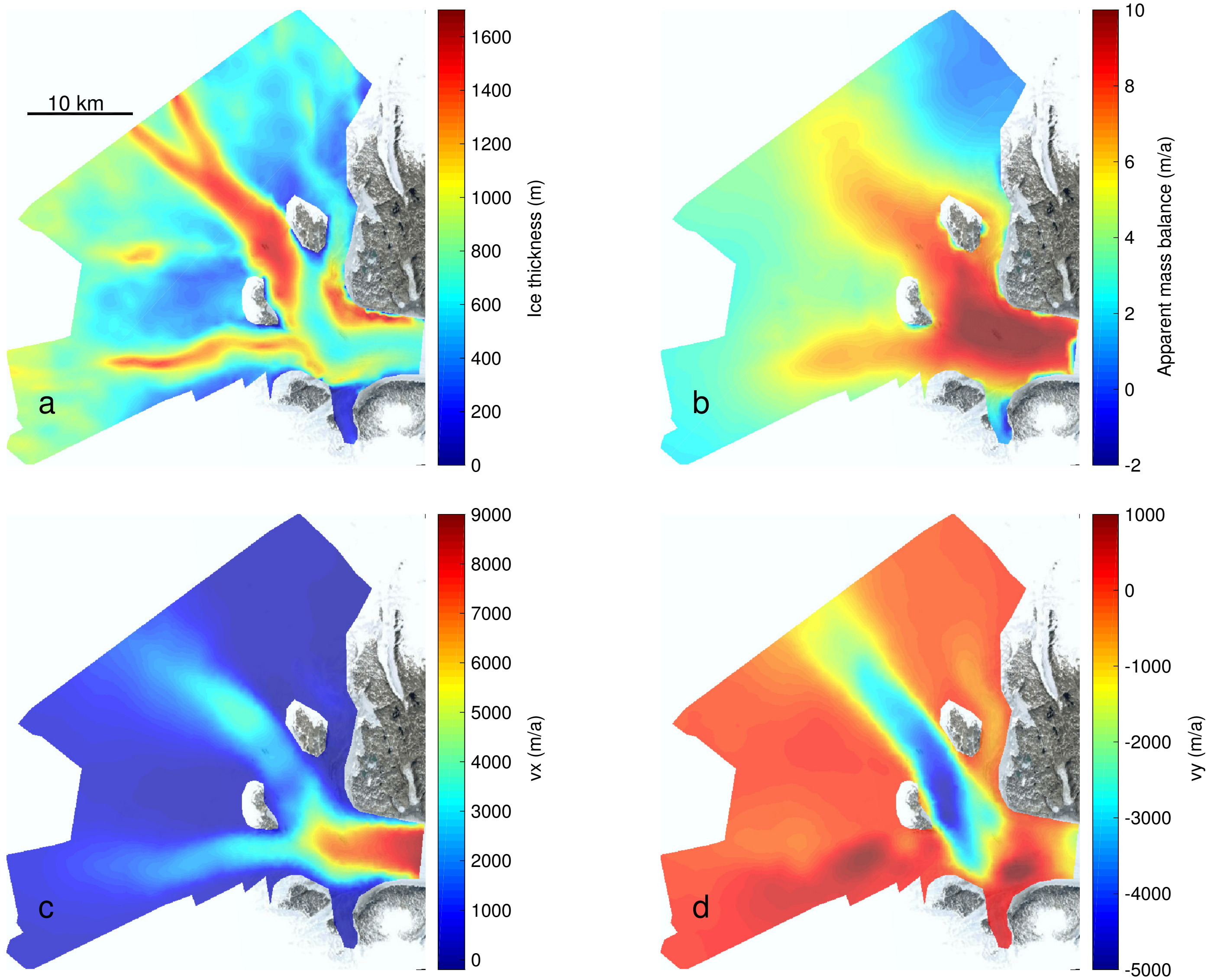

Figure S9: (a) MC calculated ice thickness before optimization, (b) initial apparent mass balance, (c) observed surface x-component of the ice velocity, (d) observed surface y-component of the ice velocity.

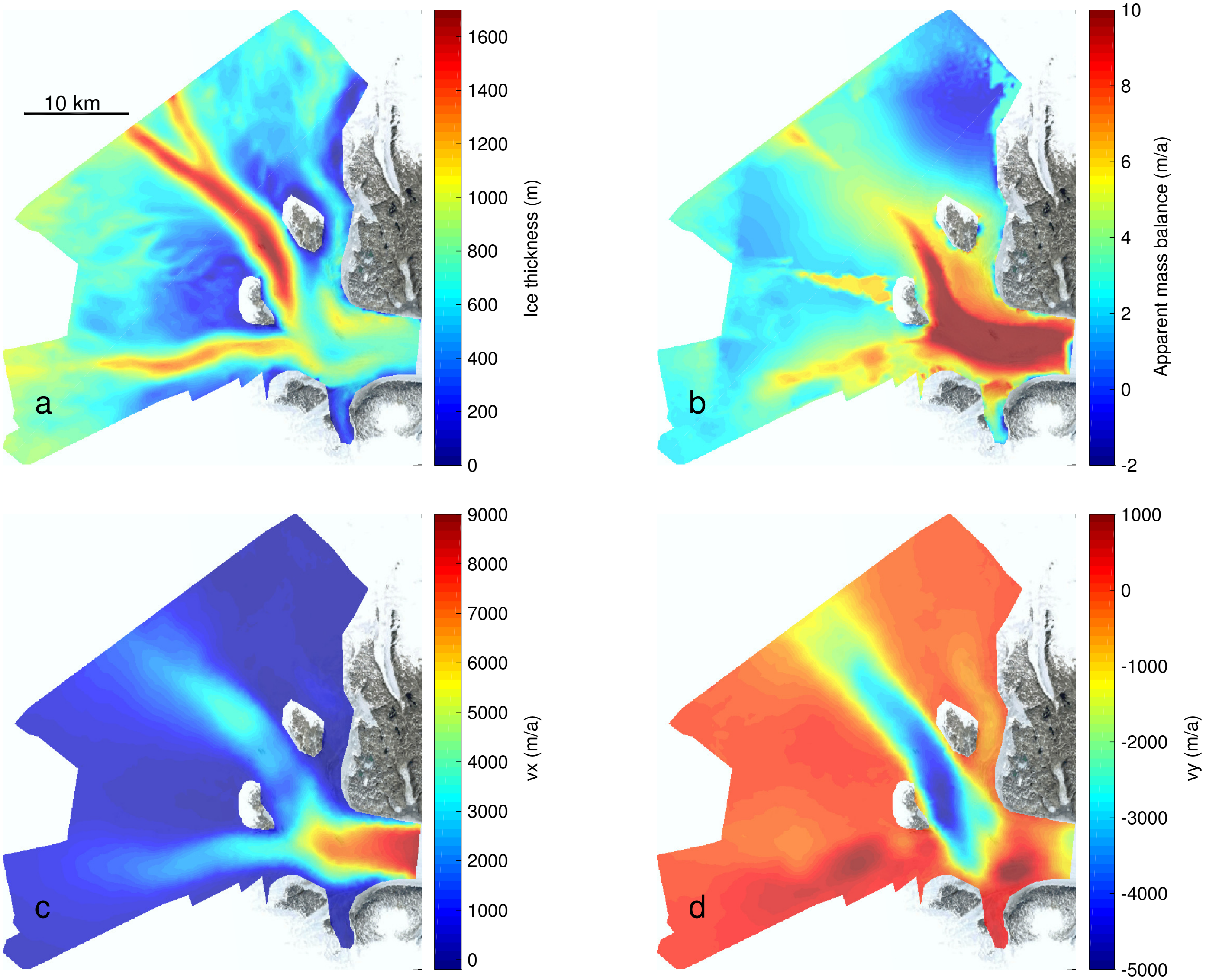

Figure S10: (a) MC calculated ice thickness after optimization, (b) optimized apparent mass balance, (c) optimized depth averaged x-component of the ice velocity, (d) optimized depth averaged y-component of the ice velocity.

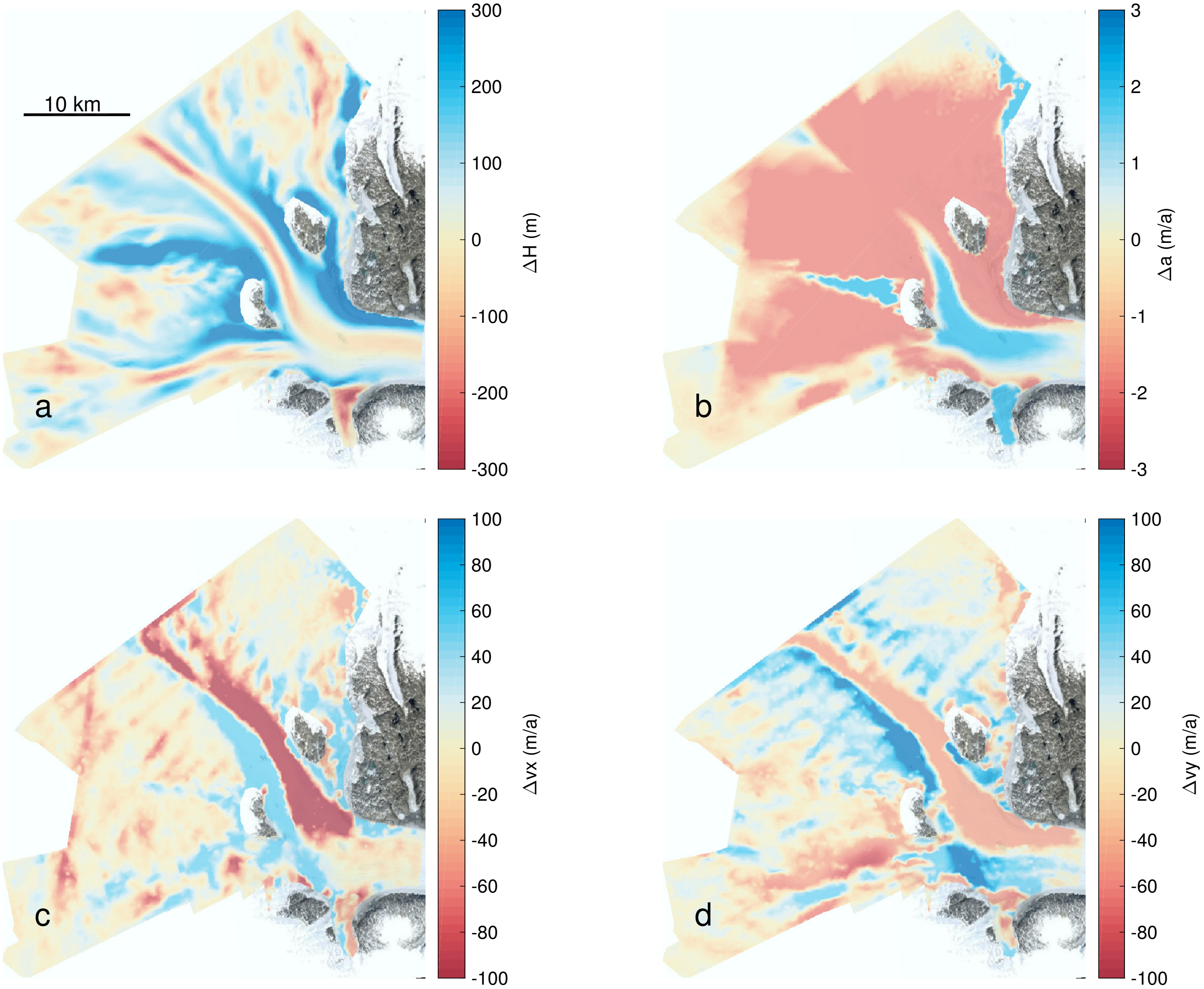

Figure S11: (a) difference between initial and optimized ice thickness, (b) difference between initial and optimized apparent mass balance, (c) between initial and optimized $\bar{v}_x$, (d) between initial and optimized $\bar{v}_y$.

References

Bamber, J. L., et al., A new bed elevation dataset for Greenland, *Cryosphere*, *7*, 499–510, doi:10.5194/tc-7-499-2013, 2013.

Howat, I. M., A. Negrete, and B. E. Smith, The Greenland Ice Mapping Project (GIMP) land classification and surface elevation datasets, *Cryosphere*, *8*(4), 1509–1518, doi:10.5194/tc-8-1509-2014, 2014.

Williams, C. N., et al., Generating synthetic fjord bathymetry for coastal Greenland, *Cryosphere*, *11*(1), 363–380, doi:10.5194/tc-11-363-2017, 2017.
