# Supplementary material for: BedMachine v3: Complete Bed Topography and Ocean Bathymetry Mapping of Greenland From Multibeam Echo Sounding Combined With Mass Conservation
Source: Geophys Res Lett. 2017 Nov 1;44(21):11051–61. doi: 10.1002/2017GL074954 (PMC5726375; doi:10.1002/2017GL074954)
Supplement: Supplementary file 2 — Table S1 [file GRL-44-11051-s002.pdf]

	Glacier name[1]	Lat	Lon	Speed (m/yr)	Drainage Area (km^2)	Glacier type	MC	Bathy	Ice Front depth below SL (m) B2013	Ice Front depth below SL (m) RTopo2	Ice Front depth below SL (m) This study	Continuously connected below 300 m B2013	Continuously connected below 300 m RTopo2	Continuously connected below 300 m This study	Continuously connected below 200 m B2013	Continuously connected below 200 m RTopo2	Continuously connected below 200 m This study	Bathymetry data coverage [3]
1	Jakobshavn Isbræ	69.158	-49.658	13255	90135.0	TW	1	1	-747.16	-851.11	-814.91	1	1	0	1	1	1	L
2	Kangerlussuaq Gletscher	68.609	-32.97	8141	95103.0	TW	1	0	-651.26	-676.16	-901.11	1	0	1	1	0	1	L
3	Helheimgletscher	66.368	-38.213	7492	50023.0	TW	1	1	-832.51	-648.03	-658.45	1	0	0	1	1	1	L
4	Køge Bugt C	65.17	-41.164	5240	18276.3	TW	1	0	-497.09	-278.53	-328.14	0	0	0	0	0	1	L
5	Rink Isbræ	71.759	-51.606	4181	33171.0	TW	1	1	-849.78	-780.04	-726.1	1	0	1	1	1	1	G
6	Store Gletscher	70.401	-50.549	3678	30466.0	TW	1	1	82.808	-219.04	-357.8	0	0	1	0	0	1	G
7	Gyldenløve Fjord C	64.245	-41.588	3519	2456.6	TW	1	0	-15.246	-63.31	-511.19	0	0	1	0	0	1	N
8	Daugaard-Jensen	71.913	-28.615	3513	49230.0	TW	1	1	-66.002	-407.83	-550.78	0	1	1	0	1	1	L
9	Umiivik Fjord	64.489	-40.677	3492	1381.9	TW	0	0	-505.25	-221.75	-85.077	1	0	0	1	0	0	N
10	Unnamed Uunartit Islands	67.431	-33.528	3371	3099.4	TW	1	0	-10.235	-147.16	-168.65	0	0	0	0	0	0	N
11	Unnamed Deception Ø CN	67.624	-33.399	3368	7305.2	TW	1	0	-5.9224	-314.7	-449.65	0	0	0	0	0	0	N
12	A.P. Bernstorff Gletscher	63.843	-41.734	3162	4399.5	TW	1	0	16.438	15.329	-458.75	0	0	0	0	0	0	L
13	Sermeq Kujalleq	69.993	-50.175	3056	20667.0	TW	1	1	-81.362	-527.17	-448.94	0	0	1	0	0	1	G
14	Køge Bugt S	64.991	-41.237	3056	3248.6	TW	1	0	-34.396	-141.01	-351.88	0	0	0	0	0	1	L
15	Tingmiarmiut Fjord	62.742	-43.339	2918	4851.7	TW	1	0	-26.046	-151.59	-383.24	0	0	0	0	0	1	L
16	Kong Oscar Gletscher	76.018	-59.697	2910	23485.0	TW	1	0	-836.18	-671.53	-670.02	0	0	1	1	0	1	L
17	Alison Gletscher	74.614	-56.081	2905	14539.0	TW	1	0	-362.99	-525.13	-472.54	1	0	0	1	0	0	N
18	Upernavik Isstrøm C	72.936	-54.293	2899	13098.6	TW	1	1	-82.518	-746.45	-618.23	0	0	1	0	0	1	L
19	Graulv / Gyldenløve Fjord N	64.354	-41.543	2805	6238.4	TW	1	0	-392.39	-72.366	-435.01	0	0	0	0	0	0	N
20	Ukaasorsuaq	61.958	-48.721	2774	12134.0	TW	1	0	42.109	-216.51	-168	0	0	0	0	0	0	VL
21	Illullip Sermia	74.405	-56.049	2666	22310.0	TW	1	1	-586.67	-444.24	-382.58	1	0	1	1	0	1	G
22	Upernavik Isstrøm S	72.848	-54.31	2546	8011.1	TW	1	1	-385.59	-298.51	-410.47	0	0	1	1	0	1	L
23	Unnamed Mogens Heinesen S	62.339	-43.114	2498	1987.7	TW	1	1	-24.801	19.753	-528.29	0	0	0	0	0	1	G
24	Unnamed south Hayes M	74.788	-56.567	2352	9258.8	TW	1	1	-51.415	-539.29	-440.79	0	0	1	0	0	1	G
25	Sermeq Silarleq	70.82	-50.775	2321	10830.0	TW	1	1	83.957	-321.69	-343.85	0	0	0	0	0	0	G
26	Upernavik Isstrøm N	73.073	-54.166	2318	22083.0	TW	1	0	-521.12	-762.55	-854.14	0	0	1	0	0	1	L
27	Steenstrup Gletscher	75.289	-57.917	2222	8537.0	TW	1	1	-174.36	-298.8	-307.25	0	0	0	1	0	1	G
28	Sverdrup Gletscher	75.587	-58.15	2156	12625.0	TW	1	1	-517.16	-422.06	-479.4	0	0	1	0	0	1	G
29	Ikertivaq N	65.633	-39.726	2131	4031.6	TW	1	1	-474.62	-261.41	-212.83	0	0	0	0	0	1	G
30	Qajuuttap Sermia	61.372	-45.734	2028	5398.0	TW	1	0	31.879	30.436	73.902	0	0	0	0	0	0	VL
31	Unnamed Mogens Heinesen N	62.549	-43.1	1999	1358.4	TW	1	0	7.4409	43.244	-246.7	0	0	0	0	0	1	G
32	Sermeq Avannarleq	69.349	-50.343	1919	613.0	TW	1	1	-920.1	-60.186	-8.8448	0	0	0	0	0	0	N
33	Unnamed Anorituup Kangerlua N	61.629	-43.116	1913	2509.8	TW	1	1	-40.03	23.522	-463.04	0	0	0	0	0	1	G
34	Unnamed Deception Ø CS	67.572	-33.467	1894	2512.6	TW	1	0	13.428	-400.04	-509.15	0	0	0	0	0	0	N
35	K.I.V. Steenstrup Nodre Bræ	66.508	-34.532	1877	3274.2	TW	0	1	-335.77	-306.44	-295.42	0	0	0	0	0	0	G
36	Kangiata Nunaata Sermia	64.261	-49.557	1860	19583.0	TW	1	0	33.82	-169.07	-148.33	0	0	0	0	0	0	L
37	Hayes Gletscher	74.944	-57.057	1857	7827.3	TW	1	1	-27.343	-263.85	-343.56	0	0	1	0	0	1	G
38	Frederiksborg Gletscher	68.261	-31.551	1824	2338.0	TW	1	1	-0.33829	-93.873	-22.548	0	0	0	0	0	0	G
39	Heimdal Gletscher	62.861	-42.616	1790	3064.2	TW	1	1	-403.02	-135.1	-161.91	1	0	0	1	0	0	G
40	Ikertivaq S	65.516	-40.208	1776	2265.6	TW	1	0	45.332	95.645	54.312	0	0	0	0	0	0	N
41	Kakivfaat Sermiat	73.489	-55.427	1760	13822.0	LT	1	1	-406.99	-595.8	-626.88	1	0	1	1	0	1	G
42	Kong Christian IV Gletscher	68.384	-30.082	1754	11090.0	LT	1	1	-514.76	-269.1	-256.43	0	0	0	1	1	1	G
43	Unnamed Herluf Trolle N	61.33	-43.33	1753	762.8	TW	1	1	-25.574	47.765	-299.42	0	0	0	0	0	0	G
44	Magga Dan Gletscher	69.952	-27.215	1692	4156.0	TW	1	0	13.502	-262.95	-126.87	0	0	0	0	0	0	N
45	Unnamed Herluf Trolle S	61.209	-43.308	1685	506.5	LT	1	1	70.131	98.291	-518.45	0	0	0	0	0	1	G
46	Ikertivaq NN	65.679	-39.657	1675	2197.0	TW	1	1	-62.571	110	139.95	0	0	0	0	0	0	VL
47	Kangerlussuup Sermersua	71.462	-51.318	1649	4979.0	TW	1	1	-613.45	-282.75	-196.1	1	0	0	1	1	0	G
48	Vestfjord Gletscher	70.369	-29.172	1628	10016.0	TW	1	0	-624.73	-228.56	-77.186	1	1	0	1	1	0	L
49	Fenrisgletscher	66.364	-37.554	1614	4967.7	TW	1	1	-754.24	-449.76	-556.94	1	0	0	1	1	0	L
50	Køge Bugt N	65.196	-40.712	1605	2443.1	TW	1	1	15.854	-32.579	-20.222	0	0	0	0	0	0	G
51	Zachariae Isstrøm	78.915	-20.535	1583	95103.0	TW	1	0	-579.6	-461.79	-580.85	0	0	0	1	1	1	N
52	Kangilerngata Sermia	69.899	-50.347	1533	7940.0	TW	1	1	16.889	-188.74	-289.94	0	0	0	0	0	1	G
53	Sermeq Avannarleq	70.061	-50.319	1523	7801.0	TW	1	1	-49.342	-252.03	-293.53	0	0	0	0	0	1	G
54	Ikertivaq M	65.597	-40.021	1519	6818.6	TW	1	1	-131.09	34.493	77.174	0	0	0	0	0	0	VL
55	Puisortoq N	62.059	-42.534	1515	1662.3	TW	1	1	1.217	107.42	-745.1	0	0	0	0	0	0	G
56	Nunatakassaap Sermia	73.228	-55.116	1505	4395.0	TW	1	1	-276.22	-262.08	-206.04	0	0	0	1	0	1	G
57	Kruuse Fjord	67.219	-33.809	1502	1133.7	TW	1	0	-13.232	-99.338	-79.998	0	0	0	0	0	0	N
58	Rimfaxe	63.233	-42.215	1481	2893.3	TW	1	1	3.0241	-184.17	-236.04	0	0	0	0	0	1	G
59	Tracy Gletscher	77.647	-66.117	1469	2967.0	TW	1	1	-720.12	-430.51	-347.37	0	0	0	0	1	0	G
60	Unnamed Mogens Heinesen C	62.49	-43.063	1459	589.6	TW	1	1	-11.04	15.059	-330.91	0	0	0	0	0	1	G
61	Polaric Gletscher	67.877	-32.497	1445	1261.0	LT	1	1	-192.59	-212.63	-283.53	0	0	0	0	0	1	G
62	Nansen Gletscher	75.738	-58.91	1441	9704.0	TW	1	1	-322.5	-302.95	-433.19	1	0	1	1	0	1	G
63	Narsap Sermia	64.638	-50.018	1408	6305.0	LT	1	0	7.2791	-148.65	-199.59	0	0	0	0	0	0	L
64	Gyldenløve Fjord S	64.165	-41.491	1386	422.5	LT	1	0	-44.052	27.355	28.365	0	0	0	0	0	0	N
65	Unnamed Laube S	66.721	-34.318	1350	691.1	LT	0	1	163.99	119.93	121.62	0	0	0	0	0	0	G
66	Qooqqup Sermia	61.203	-45.183	1304	2013.0	LT	1	0	83.865	-282.65	-299.32	0	0	0	0	0	0	L
67	Ussing Bræer	73.832	-55.612	1290	10002.0	TW	1	1	-6.0073	-437.91	-380.85	0	0	1	0	0	1	G
68	Umiammakku Isbræ	71.744	-52.393	1281	2296.0	TW	1	1	-498.49	-278.36	-378.77	0	0	0	0	0	1	G
69	Heilprin Gletscher	77.535	-66.029	1269	8760.0	TW	1	1	-242.09	-196.61	-207.92	0	0	0	0	0	0	G
70	Kangilleq	70.724	-50.618	1217	2414.0	TW	1	1	167.57	-65.203	-248.38	0	0	0	0	0	0	G
71	Gade Glacier	76.36	-62.806	1214	2958.0	TW	1	1	-222.08	-501.05	-363.37	0	0	1	0	0	1	G
72	F. Graae Gletscher	72.119	-28.713	1212	4958.2	TW	1	1	-356.76	17.996	-440.92	0	0	0	0	0	0	G
73	Mælkevejen	63.727	-41.677	1210	911.9	TW	1	0	-3.1992	31.752	-63.78	0	0	0	0	0	0	VL
74	Nioghalvfjerdsfjorden (79North)	79.331	-22.585	1199	103278.0	TW	1	0	-599.46	-591.51	-577.99	0	0	0	1	1	0	N
75	Dietrichson Gletscher	75.456	-57.99	1189	8537.0	TW	1	1	-113.13	-244.11	-334.87	0	0	0	0	0	1	G
76	Eqalorutsit Killiit Sermiat	61.289	-46.185	1172	2797.0	LT	1	0	127.16	33.964	32.475	0	0	0	0	0	0	VL
77	Køge Bugt SS	64.847	-41.09	1165	528.4	TW	1	0	-38.51	52.119	61.556	0	0	0	0	0	0	N
78	Petermann Gletscher	80.571	-60.096	1103	73817.0	TW	1	1	-449.71	-495.66	-504.18	1	1	1	1	1	1	G
79	Fimbulgletscher	63.884	-41.486	1090	378.0	LT	0	1	3.9381	39.313	47.652	0	0	0	0	0	0	G
80	Unnamed Polaric S	67.77	-33.391	1080	550.2	LT	1	0	81.443	123.88	187.27	0	0	0	0	0	0	N
81	Unnamed Anorituup Kangerlua C	61.591	-43.15	1075	517.9	TW	1	1	66.913	156.03	81.189	0	0	0	0	0	0	G
82	Skinfaxe	63.272	-41.889	1057	2451.2	LT	1	1	-376.49	-116.89	-178.63	1	0	0	1	0	0	G
83	Kjer Gletscher	75.132	-57.805	1053	6430.0	TW	1	1	-355.87	-407.18	-311.48	0	0	0	1	0	1	G
84	Unnamed Deception Ø N	67.846	-32.625	1046	856.6	TW	1	1	-29.431	13.43	30.355	0	0	0	0	0	0	N
85	Puisortoq S	61.912	-42.615	1035	706.3	TW	1	0	-26.3	16.407	-359.35	0	0	0	0	0	0	L
86	Unnamed Kangerluluk	61.111	-43.89	1034	560.7	TW	0	1	414.1	600.27	512.63	0	0	0	0	0	0	G

	Glacier name[1]	Lat	Lon	Speed (m/yr)	Drainage Area (km^2)	Glacier type	MC	Bathy	Ice Front depth below SL (m)			Continuously connected below 300 m			Continuously connected below 200 m			Bathymetry data coverage [3]
									B2013	RTopo2	This study	B2013	RTopo2	This study	B2013	RTopo2	This study	
87	Unnamed Kanger W	68.548	-32.93	1018	529.4	TW	1	1	-506.29	-311.34	-390.45	1	0	1	1	0	1	L
88	Inngia Isbræ	72.033	-52.611	980	4099.0	TW	1	1	-174.23	-74.016	-116.57	0	0	0	0	0	0	G
89	Døcker Smith Gl. W	76.336	-61.92	969	1036.0	TW	1	1	-441.98	-365.66	-531.87	1	1	0	1	1	1	G
90	Eqip Sermia	69.789	-50.239	958	1258.0	TW	1	1	36.699	-114.96	-68.234	0	0	0	0	0	0	G
91	Borggraven	68.614	-28.077	935	1138.0	TW	1	0	-48.483	3.6829	5.0843	0	0	0	0	0	0	N
92	Sermiligaarssuk Bræ	61.587	-48.323	932	5730.0	TW	1	0	25.352	52.485	55.241	0	0	0	0	0	0	L
93	Storbræ N	68.958	-25.936	893	362.0	TW	1	0	-42.51	19.965	-27.602	0	0	0	0	0	0	N
94	Gerard de Geer Gletscher	73.505	-27.266	885	3215.8	TW	1	0	-89.328	-144.81	-27.712	0	0	0	0	0	0	N
95	Avannarleq Bræ	62.227	-48.953	884	-295.4	TW	1	0	114.38	66.293	55.59	0	0	0	0	0	0	N
96	Unnamed south Hayes N	74.866	-56.763	876	4958.4	TW	1	1	10.777	-311.24	-315.75	0	0	1	0	0	1	G
97	Issuuarsuit Sermia	76.048	-60.637	875	5273.0	TW	1	1	-805.45	-413.51	-465.3	0	0	1	0	0	1	G
98	Unnamed near Upernavik	73.036	-54.545	872	2413.0	TW	1	1	-166.93	-71.072	-296.37	0	0	0	0	0	1	G
99	Rolige Gletscher	70.579	-28.295	869	12042.0	TW	1	1	-10.362	-364.03	-253.95	0	0	0	0	0	0	L
100	Døcker Smith Gletscher	76.25	-61.782	865	1938.0	TW	1	1	-454.06	-408.75	-540.52	1	1	0	1	1	1	G
101	Qeqertarsuup Sermia	73.583	-55.54	809	4087.0	TW	1	1	42.026	-301.07	-254.72	0	0	0	0	0	1	L
102	Kronborg Gletscher	68.505	-28.628	809	2335.0	TW	1	0	135.4	136.53	98.044	0	0	0	0	0	0	N
103	Melville Gletscher	77.75	-66.616	805	1099.7	TW	1	1	-301.94	26.311	-424.31	0	0	0	0	0	0	G
104	Unnamed Anorituup Kangerlua S	61.504	-42.996	798	447.2	TW	0	1	-9.3821	84.428	108.41	0	0	0	0	0	1	G
105	Unnamed south Hayes SS	74.702	-56.335	790	6619.9	TW	1	1	25.745	-190.23	-342.27	0	0	1	0	0	1	VL
106	Ussing Bræer N	73.935	-55.702	771	5000.0	TW	1	1	-87.966	-428.19	-449.94	0	0	1	0	0	1	G
107	Unnamed north Kong Oscar	76.08	-60.139	767	1807.0	TW	1	1	-105.5	-284.19	-283.21	0	0	0	0	0	1	G
108	Unnamed Danell Fjord	60.937	-43.545	749	456.7	TW	1	0	11.094	36.513	-102.45	0	0	0	0	0	1	G
109	C.H. Ostenfeld Gletscher	81.582	-45.201	740	9360.0	TW	1	0	-263.56	-226.34	-276.84	0	0	0	0	0	0	N
110	Sermilik Bræ	61.024	-46.926	739	1978.7	TW	0	0	36.244	8.3525	10.782	0	0	0	0	0	0	N
111	Hagen Bræ	81.446	-27.615	737	6506.5	LT	1	0	-264.85	-260.79	-160.53	0	0	0	0	0	0	N
112	Savissuaq Gletscher	76.318	-65.609	710	514.9	TW	1	1	-46.343	-15.736	-340.64	0	0	0	0	0	1	G
113	Berlingske Gletscher	76.993	-69.518	692	295.7	TW	1	0	-8.4348	10.331	11.623	0	0	0	0	0	0	N
114	Yngvar Nielsen Bræ	76.344	-64.084	681	780.3	LT	1	1	-211.93	-231.36	-306.16	0	0	1	0	0	1	G
115	Akullersuup Sermia	64.359	-49.572	666	1712.0	TW	1	0	49.038	-237.49	-55.902	0	0	0	0	0	0	VL
116	Hayes Gletscher N	75.024	-57.627	628	1707.0	TW	1	1	-603.47	-272.96	-324.93	1	0	0	1	0	1	G
117	Heim Gletscher	65.986	-38.5	617	443.8	LT	0	1	74.224	162.51	184.59	0	0	0	0	0	0	G
118	Carlos Gletscher	76.423	-63.431	609	126.5	TW	1	1	34.933	-196.64	-244.21	0	0	0	0	0	1	G
119	Sermilik	70.639	-50.583	583	1581.9	TW	1	1	182.44	-34.78	-37.728	0	0	0	0	0	0	G
120	Unnamed Napasorsuaq S	61.685	-42.666	571	51.5	TW	0	0	-2.255	96.045	92.467	0	0	0	0	0	0	VL
121	Midgårdgletscher	66.413	-36.825	565	2810.1	TW	1	1	22.049	-121.4	88.005	0	0	0	0	0	0	N
122	Unnamed Napasorsuaq C	61.744	-42.897	553	185.5	TW	1	0	-38.109	23.043	16.766	0	0	0	0	0	0	L
123	Unnamed Mogens Heinesen SSS	62.266	-42.574	550	295.4	TW	0	1	-6.275	0.012836	-0.29373	0	0	0	0	0	0	G
124	Bredegletscher	70.237	-25.213	543	1003.0	TW	1	1	-26.261	-0.52362	-12.921	0	0	0	0	0	0	G
125	Upernavik SS	72.782	-54.227	541	22271.6	TW	1	1	-51.309	-66.478	-204.26	0	0	0	0	0	1	G
126	Nordenskiöld Gletscher	75.834	-59.073	533	3540.0	TW	1	1	-92.125	-270.36	-258.4	0	0	0	0	0	1	VL
127	Glacier de France	66.44	-35.93	519	1083.1	TW	0	0	-36.245	38.981	45.131	0	0	0	0	0	0	N
128	Unnamed Anorituup Kangerlua SS	61.443	-42.647	518	132.8	TW	0	1	-5.1582	67.392	76.529	0	0	0	0	0	0	G
129	Dendritgletscher	69.297	-25.202	515	2381.0	TW	1	0	-12.529	8.8181	-175.84	0	0	0	0	0	0	N
130	Cornell Gletscher	74.239	-56.078	500	2255.0	TW	1	1	-53.641	-435.25	-288.24	0	0	1	0	0	1	G
131	Storbræ S	68.873	-26.102	496	356.0	TW	1	0	-54.411	-9.9642	-69.437	0	0	0	0	0	0	N
132	Kista Dan Gletscher	69.957	-27.43	482	2203.0	TW	1	0	-26.637	-224.99	-124.27	0	0	0	0	0	0	N
133	Yngvar Nielsen Bræ W	76.368	-64.362	474	537.8	TW	1	1	-976.19	-178.65	-203.2	0	0	0	0	0	0	G
134	Lille Gletscher	70.523	-50.525	471	1815.2	TW	1	1	12.079	-12.857	-157.58	0	0	0	0	0	0	G
135	Farquhar Gletscher	77.724	-66.228	463	389.0	TW	1	1	-53.128	-59.549	-46.861	0	0	0	0	0	0	G
136	Sydbræ	70.19	-26.321	445	840.0	TW	0	0	-69.802	-5.7725	-8.9422	0	0	0	0	0	0	VL
137	Rink Gletscher	76.254	-60.893	432	2661.0	TW	1	1	-50.185	-0.94697	-357.67	0	0	1	0	0	1	G
138	Styrtegletscher	68.663	-32.412	426	478.0	TW	1	0	306.84	291.41	378.64	0	0	0	0	0	0	L
139	Humboldt Gletscher	79.706	-64.27	424	47310.0	TW	1	0	-205.42	-222.28	-216.73	0	0	0	1	1	1	N
140	Unnamed Napasorsuaq N	61.842	-42.846	421	495.9	TW	1	1	25.672	114.31	-248.21	0	0	0	0	0	1	G
141	Unnamed Kanger E	68.525	-32.867	411	5130.5	TW	1	1	-226.66	-62.564	-41.033	1	0	0	1	0	0	VL
142	Frederikshåbs Isblink	62.458	-50.051	411	1523.0	TW	1	0	93.402	78.867	84.339	0	0	0	0	0	0	N
143	Unnamed South Danell Fjord	60.849	-43.623	407	196.4	TW	1	1	89.829	57.493	-191.52	0	0	0	0	0	1	G
144	Ryder Gletscher	81.473	-50.276	405	142.2	TW	1	0	-807.15	-849.21	-875.83	1	1	1	1	1	1	N
145	Bowdoin Gletscher	77.678	-68.63	401	29788.0	TW	0	1	-5.5159	-2.083	-4.5259	0	0	0	0	0	0	G
146	Savissuaq W	76.29	-66.086	400	1015.1	TW	1	1	77.052	-5.0782	-262.58	0	0	0	0	0	0	G
147	Unnamed Mogens Heinesen SS	62.392	-42.717	395	196.0	TW	0	1	16.666	54.154	49.989	0	0	0	0	0	0	G
148	Unnamed south Russell	66.984	-50.012	383	183.1	TW	1	0	44.138	81.414	75.443	0	0	0	0	0	0	X
149	Hubbard Gletscher	77.603	-67.742	376	261.2	TW	0	1	-5.4963	-3.3716	-7.8373	0	0	0	0	0	0	G
150	Savissuaq WWWW	76.191	-67.539	363	9930.3	TW	1	1	-17.817	-47.915	-215	0	0	0	0	0	0	G
151	Carlos Gletscher W	76.393	-63.531	347	258.1	TW	1	1	-24.033	-8.5466	-6.2929	0	0	0	0	0	0	G
152	Laube Gletscher	66.925	-34.092	345	424.9	TW	1	0	144.27	71.599	68.641	0	0	0	0	0	0	N
153	Knud Rasmussen	66.088	-36.312	340	682.3	TW	0	0	3.7931	0.39717	28.549	0	0	0	0	0	0	VL
154	Sorgenfri Glacier	68.307	-30.828	326	63.8	TW	1	0	-26.922	-87.395	-43.262	0	0	0	0	0	0	N
155	Adolf Hoel Gletscher	73.951	-25.838	309	457.3	TW	1	1	-8.6583	-252.95	12.393	0	0	0	0	0	0	N
156	Sharp Gletscher	77.702	-66.96	287	729.0	TW	1	0	70.697	13.142	0.57917	0	0	0	0	0	0	G
157	Unnamed Vestfjord S	70.005	-27.977	286	11118.8	TW	1	0	-54.494	-166.91	-47.62	0	0	0	0	0	0	N
158	K.I.V. Steenstrup, Søndre Bræ	66.469	-34.697	282	259.3	TW	0	0	-27.677	9.4657	23.269	0	0	0	0	0	0	L
159	Steensby Gletscher	81.468	-54.413	276	780.8	TW	1	0	-36.042	-83.631	-92.622	0	0	0	0	0	0	N
160	Unnamed Dendritgletscher S	69.233	-25.439	268	212.1	TW	1	0	-32.229	1.6371	-15.572	0	0	0	0	0	0	N
161	Rosenborg Gletscher	68.509	-28.975	266	3735.0	TW	1	0	74.534	90.502	43.825	0	0	0	0	0	0	N
162	Torvgletscher	69.949	-23.027	262	180.0	TW	0	0	-13.093	26.414	45.097	0	0	0	0	0	0	N
163	Academy Gletscher	81.628	-32.381	261	919.0	TW	1	0	-293.77	-212.08	-215.95	0	0	0	0	0	0	N
164	Qooqqup Sermia S'	61.197	-45.055	260	410.0	TW	1	0	187.5	142.62	-12.989	0	0	0	0	0	0	L
165	Unnamed south bis Russell	66.951	-49.96	258	57.0	TW	1	0	209.01	175.17	158.22	0	0	0	0	0	0	X
166	Kangilinnguata Sermia	64.837	-49.946	258	3539.2	TW	0	0	118.75	150.54	121.9	0	0	0	0	0	0	N
167	Courtauld Gletscher	68.511	-32.205	252	25278.8	TW	1	1	68.322	54.313	48.698	0	0	0	0	0	0	VL
168	Saqqarliup Sermia	68.895	-50.324	251	57.0	TW	1	0	10.696	-104.77	-144.04	0	0	0	0	0	0	L
169	Brikkerne Gletscher	81.816	-45.618	241	9930.3	TW	1	0	-43.193	-31.751	-89.892	0	0	0	0	0	0	N
170	Nigerlikasik	62.079	-48.756	231	3784.0	TW	1	0	126.17	80.536	129.11	0	0	0	0	0	0	N
171	Savissuaq WWW	76.253	-67.297	229	263.0	TW	1	1	24.015	-104.23	-232.08	0	0	0	0	0	1	G
172	Sioralik Bræ	61.522	-48.14	213	544.0	TW	1	0	75.865	66.819	65.27	0	0	0	0	0	0	X
173	Qamanaarsuup Sermia	64.465	-49.629	211	1518.7	TW	1	0	61.889	-33.727	33.532	0	0	0	0	0	0	X
174	Sortebræ	68.765	-27.042	210	2450.6	TW	0	0	-5.7211	70.732	64.513	0	0	0	0	0	0	N

	Glacier name[1]	Lat	Lon	Speed (m/yr)	Drainage Area (km^2)	Glacier type	MC	Bathy	Ice Front depth below SL (m) B2013	Ice Front depth below SL (m) RTopo2	Ice Front depth below SL (m) This study	Continuously connected below 300 m B2013	Continuously connected below 300 m RTopo2	Continuously connected below 300 m This study	Continuously connected below 200 m B2013	Continuously connected below 200 m RTopo2	Continuously connected below 200 m This study	Bathymetry data coverage [3]
175	Diebitsch Gletscher	77.942	-71.627	209	973.7	TW	0	0	28.837	31.281	37.221	0	0	0	0	0	0	N
176	Unnamed south ter Russell	66.714	-49.814	200	463.0	TW	1	0	263.18	126.8	233.7	0	0	0	0	0	0	X
177	Harder Gletscher	81.635	-44.966	197	395.5	TW	1	0	-35.461	-48.881	-23.121	0	0	0	0	0	0	N
178	South of Sermeq	63.448	-49.647	195	7207.5	TW	1	0	485.84	481.03	489.23	0	0	0	0	0	0	X
179	Helland Gletscher E	76.283	-64.624	194	177.2	TW	1	1	-105.67	-9.9327	-173.45	0	0	0	0	0	0	G
180	Thrym Gletscher	63.557	-41.776	193	1474.5	TW	1	0	-12.287	67.149	60.668	0	0	0	0	0	0	N
181	Nakkaasorsuaq	63.06	-49.688	191	1712.0	TW	1	0	212.94	124.53	129.91	0	0	0	0	0	0	N
182	Majorqaq	65.747	-50.653	188	112.1	TW	1	0	112.98	140.1	144.26	0	0	0	0	0	0	X
183	Usulluup Sermia	67.939	-50.35	187	883.5	TW	1	0	29.781	-24.967	57.698	0	0	0	0	0	0	N
184	Unnamed south Arsuk	61.026	-47.712	185	252.9	TW	0	0	40.975	75.242	57.33	0	0	0	0	0	0	X
185	Jættegletscher	73.446	-27.723	185	962.0	TW	1	0	-16.443	-182.11	5.6556	0	0	0	0	0	0	N
186	Charcot Gletscher	72.071	-29.409	177	254.0	TW	1	0	532.99	497.89	573.78	0	0	0	0	0	0	VL
187	Steno Bræ	69.869	-23.376	175	0.0	TW	0	0	23.196	36.301	46.458	0	0	0	0	0	0	X
188	Nordenskiöld Gletscher	68.304	-51.17	173	7207.5	TW	1	0	27.661	-9.8568	42.728	0	0	0	0	0	0	X
189	Saqqap Sermersua	65.211	-50.663	169	220.2	TW	1	0	-349.05	-367.42	-123.43	0	0	0	0	0	0	X
190	Wordie Gletscher	74.08	-22.549	168	67.0	TW	1	0	-24.156	-139.72	-25.81	0	0	0	0	0	0	N
191	Kangerluarsuup Sermia	71.249	-51.485	167	9930.3	TW	1	1	36.168	-34.351	-135.11	0	0	0	0	0	0	G
192	Savissuaq WW	76.319	-66.826	165	1358.0	TW	1	1	31.275	10.711	6.5655	0	0	0	0	0	0	G
193	Morell Gletscher	76.278	-62.525	160	4607.7	TW	1	1	-574.71	-113.95	-177.29	1	0	0	1	1	0	G
194	Nordenskiöld Gletscher	73.117	-28.05	157	104.6	TW	1	0	-85.575	-79.504	-160.64	0	0	0	0	0	0	N
195	Verhoeff Gletscher	77.853	-69.923	149	314.7	TW	0	0	11.893	25.463	31.599	0	0	0	0	0	0	N
196	Inuppaat Quuat	67.511	-50.001	147	2481.2	TW	1	0	103.39	110.39	125.05	0	0	0	0	0	0	X
197	Kangaasarsuup Sermia	64.111	-49.93	146	131.8	TW	0	0	262	224.1	213.36	0	0	0	0	0	0	X
198	Kaarale Gletscher	66.093	-36.691	141	5130.5	TW	0	0	-31.299	68.714	68.001	0	0	0	0	0	0	L
199	Unnamed Steenstrup, Søndre Bræ S	66.377	-35.271	137	9930.3	TW	0	0	24.972	51.375	53.625	0	0	0	0	0	0	N
200	Violingletscher	72.135	-26.578	133	2636.0	TW	1	0	38.44	7.5931	102.43	0	0	0	0	0	0	X
201	Jungersen	82.098	-42.97	124	755.0	TW	0	0	-33.593	10.237	10.53	0	0	0	0	0	0	N
202	Sermeq (glacier in Sermilik ice fjord)	63.543	-50.644	124	3237.0	TW	1	0	111.16	125.21	126.25	0	0	0	0	0	0	N
203	Wahlenberg Gletscher	72.507	-27.021	124	1474.5	TW	1	0	54.528	-15.55	208.98	0	0	0	0	0	0	X
204	Arsuk Bræ	61.301	-47.821	124	108.0	TW	0	0	327.01	114.48	118.53	0	0	0	0	0	0	L
205	Kiattuut Sermiat	61.236	-45.301	124	158.1	TW	1	0	91.861	28.216	39.454	0	0	0	0	0	0	X
206	Isunnguata Sermia	67.186	-50.311	122	1721.0	TW	1	0	-197.66	135.8	121.18	0	0	0	0	0	0	X
207	Unnamed south qua Russell	66.527	-49.592	115	420.4	TW	1	0	564.35	509.42	517.28	0	0	0	0	0	0	X
208	Unnamed Knud W	66.086	-36.554	114	24137.0	TW	0	0	119.1	106.49	84.626	0	0	0	0	0	0	L
209	Unnamed Hare Fjord	70.97	-28.345	114	1474.5	TW	1	0	109.64	-80.048	-0.74954	0	0	0	0	0	0	N
210	Morris Jesup Gletscher	77.886	-71.16	110	10832.9	TW	0	0	-26.569	18.066	24.419	0	0	0	0	0	0	N
211	Unnamed west of Ostenfeld	81.569	-45.867	106	589.1	TW	1	0	-43.664	-5.576	-20	0	0	0	0	0	0	N
212	Hisinger Gletscher	72.834	-27.579	105	503.0	TW	1	0	-4.938	-9.6962	7.0689	0	0	0	0	0	0	N
213	Russell Gletscher	67.095	-50.202	103	4569.8	TW	1	0	-14.203	227.05	253.03	0	0	0	0	0	0	X
214	Leidy Gletscher	77.26	-66.085	101	13602.0	TW	0	1	-9.1413	25.7	17.324	0	0	0	0	0	0	G
215	Waltershausen Gletscher	73.812	-24.244	100	9930.3	TW	1	1	-40.961	-83.603	-76.031	0	0	0	0	0	0	G
216	Storstrømmen	76.718	-22.339	96	4607.7	TW	0	0	-323.49	-294.23	-294.19	0	0	0	1	0	1	N
217	Helland Gletscher	76.191	-64.88	95	18356.8	TW	0	1	-18.784	2.6302	-163	0	0	0	0	0	0	G
218	Marie Sophie Gletscher	81.784	-32.872	74	1133.0	TW	1	0	-146.55	-188.28	-404.36	0	0	0	0	0	0	N
219	Knud Rasmussen Gletscher	76.712	-67.925	74	142.1	TW	1	0	50.101	24.153	-78.297	0	0	0	0	0	0	N
220	Alangorliup Sermia	68.943	-50.232	68	11533.0	TW	1	0	30.031	-23.214	27.104	0	0	0	0	0	0	N
221	Harald Moltke Bræ	76.593	-67.821	63	4607.7	TW	1	0	-34.086	-211.7	-192.11	0	0	0	0	0	0	N
222	Tvegegletscher	74.811	-22.348	62	49.0	TW	0	0	476.29	378.13	376.86	0	0	0	0	0	0	X
223	Mohn Gletscher	76.238	-63.628	59	286.1	TW	0	1	-32.055	-70.863	-88.077	0	0	0	0	0	0	G
224	Hart Gletscher	77.682	-67.146	58	1308.1	TW	1	1	123.42	12.273	6.0183	0	0	0	0	0	0	G
225	Henson Gletscher	82.262	-40.145	53	1193.0	LW	0	0	20.092	-3.6695	5.0366	0	0	0	0	0	0	N
226	Heinkel Gletscher	75.175	-22.906	48	9930.3	TW	0	0	54.706	94.801	95.437	0	0	0	0	0	0	N
227	Chamberlin Glestscher	76.691	-68.352	48	92.5	TW	1	0	30.376	12.543	6.3558	0	0	0	0	0	0	N
228	Soranerbræen Gletscher	76.193	-21.846	48	0.0	LT	1	0	-24.911	-34.692	8.1995	0	0	0	0	0	0	N
229	Dodge Gletscher	78.174	-72.676	46	407.6	LT	0	0	6.8091	35.745	49.997	0	0	0	0	0	0	X
230	Eielson Gletscher	71.137	-27.88	36	15361.2	LT	1	0	-37.96	-95.348	53.39	0	0	0	0	0	0	N
231	Pasterze	74.673	-22.546	26	963.3	LT	0	0	383.91	296.49	293.64	0	0	0	0	0	0	X
232	Unnamed Dendritgletscher N	69.483	-24.826	25	59366.5	LT	1	0	-41.993	7.3907	13.907	0	0	0	0	0	0	N
233	Nordfjord Gletscher	68.646	-32.603	22	310.9	LT	1	0	159.9	15.409	43.408	0	0	0	0	0	0	L
234	Pitugfik Gletscher	76.222	-68.84	21	2295.8	LT	0	0	-5.0381	22.625	15.007	0	0	0	0	0	0	L
235	Bartholin Bræ	69.593	-23.994	17	3050.2	LT	0	0	-17.158	28.693	38.619	0	0	0	0	0	0	N
236	Einar Mikkelsen Gletscher	75.571	-22.47	14	67.0	LT	1	0	237.95	33.877	35.983	0	0	0	0	0	0	N
237	Naravana Fjord	82.35	-41.847	14	541.0	LT	0	0	72.383	2.8726	2.9204	0	0	0	0	0	0	N
238	Storm Gletscher	78.141	-72.771	13	176.6	LT	0	0	144.38	37.668	35.134	0	0	0	0	0	0	N
239	Marie Gletscher	77.196	-66.087	11	3704.9	LT	0	0	140.63	28.994	50.439	0	0	0	0	0	0	N
240	Kofoed-Hansen Bræ	77.528	-21.833	8	2230.9	LT	1	0	-110.37	-148.27	-102.97	0	0	0	0	0	0	N
241	Newman Bugt	81.33	-57.287	7	301.0	LT	0	0	196.45	7.8675	22.741	0	0	0	0	0	0	N
242	L. Bistrup Bræ	76.641	-22.696	6	24080.0	LT	0	0	-41.519	-90.959	-125.54	0	0	0	0	0	0	N
243	Sermiarsupaluk (glacier)	77.084	-66.222	5	506.9	LT	0	0	316.96	50.03	51.763	0	0	0	0	0	0	N
	**TOTAL**				**1672850.1**	**211**	**194**	**107**				**22**	**7**	**28**	**32**	**17**	**67**	

Ice front depth only

		Number of glaciers Front below SL[2]	Number of glaciers Front depth < -200 m	Number of glaciers Front depth < -300 m
ALL GLACIERS (243 glaciers)	B2013	111	51	42
	Rtopo-2	120	69	38
	This study	134	89	59
MC ONLY (194 glaciers)	B2013	100	48	39
	RTopo-2	115	66	37
	This study	128	87	59

Ice front connected to ocean waters continuously below a certain depth

		Number of glaciers water remains < -300 m	Number of glaciers water remains < -200 m	Corresponding drainage basin (<-300 m)	Corresponding drainage basin (<-200 m)
ALL GLACIERS (243 glaciers)	B2013	22	32	26.6%	45.2%
	Rtopo-2	7	17	13.5%	34.9%
	This study	28	67	29.2%	54.7%

NOTES:

[1] List of glaciers based on Rignot and Mouginot 2012, red = constrained by MC and bathymetry data, green = constrained by MC only, white = kriging
glaciers in red are mapped with MC and include a bathymetry constraint
glaciers in green are mapped with MC but were not constrained at the ice front
glaciers in white are mapped using Kriging only
[2] Glacier front is considered below SL if bed < -25 m (B2013 has 50 glaciers whose front are betweem 0 and -25 m)
[3] X = land terminating, G = good, L = limited (significant data gaps), VL = very limited (sporadic measurements), N = no data
